# Paternal Obesity‐Induced H3K27me3 Elevation Leads to MANF‐Mediated Transgenerational Metabolic Dysfunction in Female Offspring

**DOI:** 10.1002/advs.202415956

**Published:** 2025-03-05

**Authors:** Yajun Shi, Weisheng Li, Xi Yu, Yan Zhao, Dan Zhu, Yueyang Song, Zejun Zhao, Yannan Gu, Bin Wei, Lingjun Li, Dongyi Yu, Pengjie Zhang, Qinqin Gao, Miao Sun

**Affiliations:** ^1^ Institute for Fetology First Affiliated Hospital of Soochow University Suzhou City Jiangsu 215031 China; ^2^ McKusick‐Zhang Center for Genetic Medicine State Key Laboratory for Complex Severe and Rare Diseases Institute of Basic Medical Sciences Chinese Academy of Medical Sciences School of Basic Medicine Peking Union Medical College Beijing 100005 China; ^3^ Department of Gynecology University of Health and Rehabilitation Sciences Qingdao Hospital (Qingdao Municipal Hospital) Shandong province Qingdao 266000 China; ^4^ Center for Medical Genetics and Prenatal Diagnosis Shandong Provincial Maternal and Child Health Care Hospital Affiliated to Qingdao University Shandong province Jinan 250000 China

**Keywords:** H3K27me3, MANF, paternal obesity, sperm, transgenerational inheritance

## Abstract

Paternal lifestyle and environmental exposures can alter epigenetic changes in sperm and play a critical role in the offspring's future health, yet the underlying mechanisms remain elusive. The present study established a model of paternal obesity and found that the increased levels of H3K27me3 in sperm persist into the 8‐cell embryo stage, resulting in a transgenerational decrease of *Manf*, which causes endoplasmic reticulum stress and activates the GRP78‐PERK‐EIF2α‐ATF4‐CHOP axis. This consequently leads to impaired glucose metabolism and apoptosis in the liver of female offspring. Based on these findings, the F0 mice are treated with 3‐deazaneplanocin A, an EZH2 inhibitor, which successfully prevented metabolic dysfunction in F0 mice of the high‐fat diet (HFD) group. Meanwhile, intravenous injection of recombinant human MANF in F1 female offspring can successfully rescue the metabolic dysfunction in the HFD‐F1 group. These results demonstrate that paternal obesity triggers transgenerational metabolic dysfunction through sperm H3K27me3‐dependent epigenetic regulation. The present study also identifies the H3K27me3‐MANF pathway as a potentially preventive and therapeutic strategy for diabetes, although further studies are needed to validate its clinical applicability.

## Graphical abstract

Paternal lifestyle and environmental exposures play a critical role in the offspring's health. The present study established a paternal obesity model and demonstrated that elevated H3K27me3 levels in sperm persist through the 8‐cell embryo stage. This epigenetic modification triggers transgenerational decrease of MANF, inducing ER stress and activating the GRP78‐PERK‐EIF2α‐ATF4‐CHOP axis, ultimately driving hepatic metabolic dysfunction and apoptosis in female offspring.

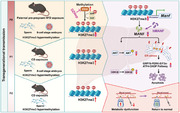

## 1 Introduction

Obesity is a severe, chronic, and complicated condition characterized by excessive fat deposits. By 2022, the global incidence of adult obesity had more than doubled since 1990, with 1 in 8 people in the world living with obesity. Of these, 160 million were adolescents aged 5–19 years, forecast to reach more than 750 million in 2035.^[^ ^1^ ^]^ If these predicted increases occur, they will undoubtedly exacerbate the economic burdens of obesity.^[^ ^2^ ^]^ Robust evidence demonstrates that obesity influences the development and functionality of numerous organs and correlates with a heightened prevalence of diabetes, hypertension, cardiovascular disease, nonalcoholic fatty liver disease, cancer, and male infertility.^[^ ^3^ ^]^ Notably, both obesity and diabetes are linked to metabolic dysregulation, fat expansion, peripheral insulin resistance (IR), and hepatic steatosis. A detailed understanding of the molecular pathways regulating glucose and lipid metabolism is essential to identify potential targets for effective treatment of obesity and diabetes.^[^ ^4^ ^]^ However, the molecular mechanisms underlying the increased incidence of diabetes in obese individuals remain largely unclear.

Previous studies in humans and animal models have established that paternal obesity‐induced epigenetic changes in sperm could be transmitted to the first generation (F1), leading to a range of diseases such as hypertension, reproductive impairment, and metabolic dysfunction, including insulin resistance, hyperleptinemia, and lipid accumulation in the ovaries of female offspring.^[^ ^5^ ^]^ Moreover, emerging studies have established that F1 male offspring from obese fathers can transmit similar phenotypes to the second generation (F2), suggesting that epigenetic information changes (such as histone modifications, DNA methylation, and sperm noncoding RNAs) can be transmitted through chromatin condensation to impact embryonic gene expression, development, and offspring health.^[^ ^5^, ^6^ ^]^ Furthermore, multiple studies have investigated that there are sex‐specific effects in offspring traits induced by paternal obesity.^[^ ^7^ ^]^ There is still some disagreement about how much epigenetic information is passed down from one generation to the next. To date, several studies that investigated the effects of a high‐fat diet (HFD) and obesity on the sperm epigenome mainly focused on DNA methylation and noncoding RNA (ncRNA) as the possible metabolic disease‐causing factors in sperm.^[^ ^8^ ^]^ The role of sperm chromatin in the non‐genetic inheritance of metabolic disorders requires further elucidation. While the vast majority of the histones are replaced by protamine in spermiogenesis, a small portion of histones are retained on gene regulatory elements in sperm, which can mirror the gene expression patterns of the next generation.^[^ ^9^ ^]^ To prevent transmission of disease from father to offspring, it is critically important to understand how histone changes in sperm affect the embryo's functionality.

Trimethylation of histone H3 at lysine 27 (H3K27me3), catalyzed by polycomb repressive complex 2 (PRC2), plays an important regulatory role in silencing critical gene expressions for embryogenesis, organogenesis, and tumorigenesis.^[^ ^9^, ^10^ ^]^ Moreover, *Arabidopsis*, *C. elegans*, and mouse models have demonstrated that H3K27me3 in sperm might be associated with offspring phenotypes.^[^ ^9^, ^10^, ^11^ ^]^ In various tissues, deletion of the enhancer of zeste homolog 2 (*Ezh2*), the catalytic component of PRC2, results in defective cellular functions due to abnormal gene expressions by decreased H3K27me3, suggesting that EZH2 is a core molecule in PRC2 that mediates H3K27 methylations.^[^ ^12^ ^]^ Despite these advancements, the function of H3K27me3 in paternal epigenetic inheritance requires further investigation. To date, the effects of paternal HFD exposure on H3K27me3 and whether it will be transmitted to offspring, resulting in metabolic disorders, remain unclear.

The liver is a vital organ in the modulation of energy homeostasis because it detects and reacts to shifts in the nutritional condition that occur in response to a wide range of metabolic circumstances, mainly through the release of hepatokines.^[^ ^13^ ^]^ Of note, mesencephalic astrocyte‐derived neurotrophic factor (MANF), a feeding‐induced hepatokine, plays an important role in maintaining normal endoplasmic reticulum (ER) homeostasis. Recent studies have shown that early‐onset and severe diabetes are the major phenotypes in both embryonic knockout of MANF globally or hepatocyte‐specific MANF knockout mice, highlighting the significant role of MANF in diabetes.^[^ ^14^ ^]^ In times of ER stress, low calcium levels in the ER cause MANF to separate from the MANF‐78 kDa glucose‐regulated protein (GRP78) complex, which helps with MANF secretion.^[^ ^15^ ^]^ Meanwhile, patients with type 2 diabetes showed lower serum levels of MANF, which correlated with the abnormal metabolism of lipids and glucose.^[^ ^16^ ^]^ Further evidence documented that obesity in mice is known to cause ER stress, which impairs protein kinase (AKT) phosphorylation (p‐AKT), contributing to peripheral insulin resistance develops.^[^ ^17^ ^]^ If prolonged, unresolved ER stress will induce cell death, which is mainly mediated by phosphorylating the eukaryotic translation initiation factor 2 subunit 1 (EIF2α), which suppresses global protein synthesis but selectively enhances the translation of activating transcription factor 4 (ATF4) and DNA damage‐inducible transcript 3 (DDIT3/CHOP).^[^ ^18^ ^]^ In fact, MANF is highly abundant in the liver, and hepatic MANF expression was found to be significantly decreased in HFD mice.^[^ ^19^ ^]^ A subsequent study showed that systemic MANF administration ameliorates insulin resistance in obese mice, highlighting its therapeutic potential.^[^ ^20^ ^]^ Therefore, MANF might be a promising target for treating chronic metabolic diseases. However, whether MANF is involved in the diabetic phenotype caused by paternal obesity is yet unclear.

Our previous studies have found that paternal HFD exposure enhances hepatic gluconeogenesis in male offspring, while their glucose tolerance and insulin sensitivity remain largely unaffected.^[^ ^21^ ^]^ In this study, we evaluated whether paternal obesity impacts metabolic phenotypes in the female F1 and F2 offspring, and explored the role of the EZH2‐H3K27me3 axis in this transgenerational inheritance. We present evidence that EZH2 represses *Manf* transcription by increasing the abundance of H3K27me3 at its promoter regions, ultimately resulting in the decrease of MANF. Reduced expression of MANF could lead to metabolic dysfunction and apoptosis, potentially contributing to diabetes in the F1. Strikingly, we observed transgenerational phenotypes in F2, which were associated with elevated H3K27me3 in paternal sperm. These suggest that the risk of transgenerational trait transmission may be higher if an ancestor has HFD exposures that cause pre‐existing damage to the sperm epigenome. As expected from the metabolic traits seen in the female offspring, changes in sperm H3K27me3, a site involved in embryonic development, apoptosis, and glucose metabolism pathways. The enhancement of H3K27me3 in sperm persists in the 8‐cell embryo, which supports the idea that sperm H3K27me3 does play a part in the ancestry of metabolic disorders that show up in adulthood.

## 2 Results

### 2.1 Paternal Obesity Induces Transgenerational Transmission of Glucose Insensitivity, Insulin Resistance, and Impaired Fertility

**Figure 1:**
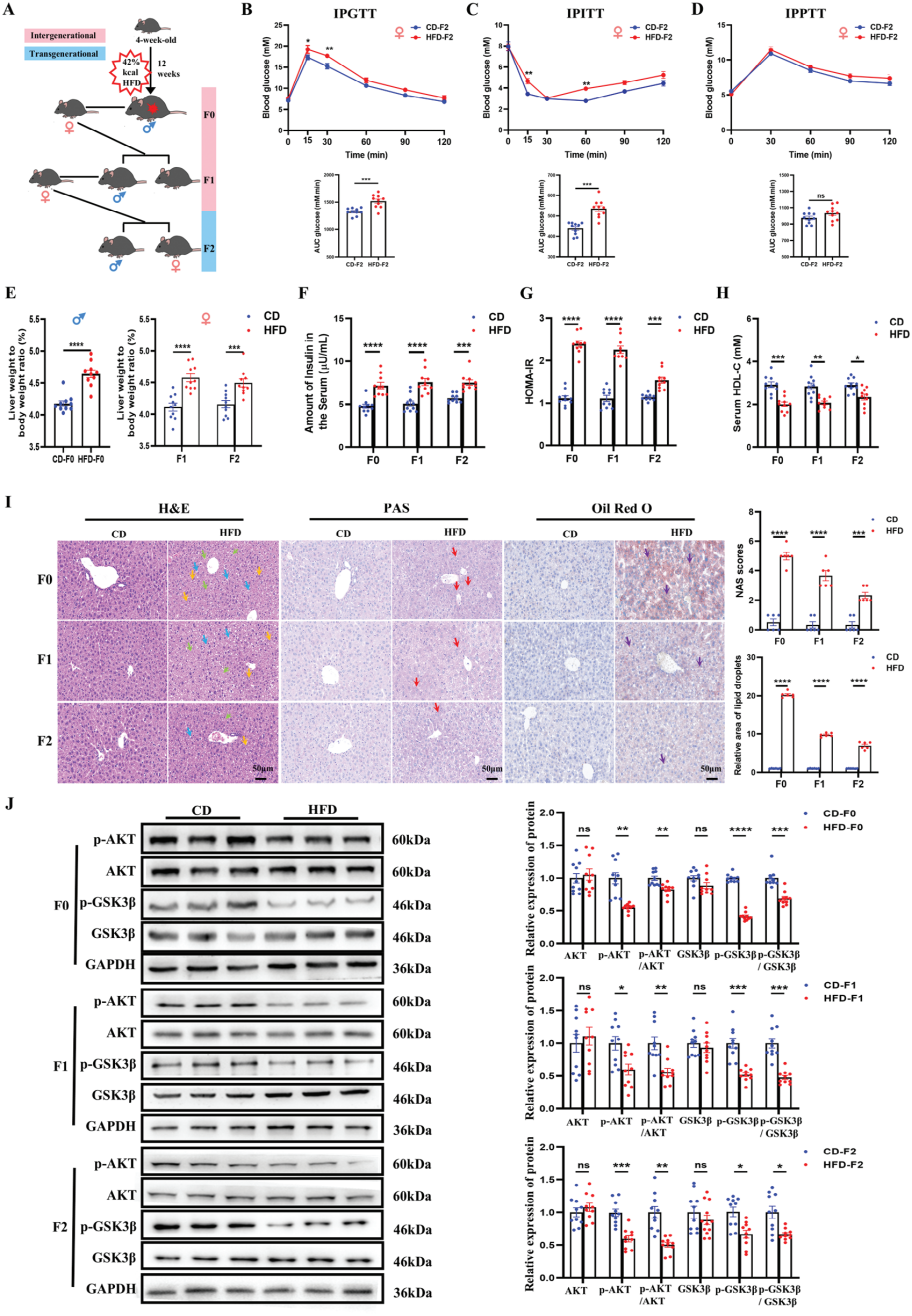
Paternal obesity induces transgenerational transmission of glucose insensitivity, insulin resistance in female offspring. A) Schematic illustration of the experimental concept for producing intergenerational and transgenerational offspring of paternal CD or HFD exposure; B) Glucose tolerance test and AUC at 22‐week‐old in female offspring of two groups (CD‐F2, n = 10; HFD‐F2, n = 10); C) Insulin tolerance test and AUC at 24‐week‐old in female offspring of two groups (CD‐F2, n = 10; HFD‐F2, n = 10); D) Pyruvate tolerance test and AUC at 26‐week‐old in female offspring of two groups (CD‐F2, n = 10; HFD‐F2, n = 10); E) Liver/body weight ratio of F0 mice and F1‐F2 female mice (n = 10 each group); F) Fasting serum insulin levels of F1–F2 (n = 10 each group); G) HOMA‐IR of F0 male mice and F1‐F2 female mice (n = 10 each group); H) Serum HDL‐C levels of F0 male mice and F1‐F2 female mice (n = 10 each group); (I) The H&E, PAS, and Oil Red O staining of F0 male mice and F1‐F2 female mice (n = 3 each group). Fat vacuoles (blue arrow), infiltration of inflammatory cells (yellow arrows), and cell swelling (green arrows), glycogen accumulation (red arrows), triglyceride accumulation (purple arrows). NAS score and lipid droplet area in different groups (n = 6 each group); J) Protein levels of AKT, p‐AKT, p‐AKT/AKT, GSK3β, p‐GSK3β, and p‐GSK3β/GSK3β in liver of F0 male mice and F1‐F2 female mice from CD and HFD group (n = 10 each group). Data are mean ± SEM. ns, no significance; ^*^ *p* < 0.05; ^**^ *p* < 0.01; ^***^ *p* < 0.001; ^****^ *p* < 0.0001 (two‐tailed t‐test or one‐way/two‐way ANOVA).

A growing amount of research has demonstrated a significant correlation between paternal obesity and offspring metabolic phenotypes.^[^ ^8^, ^21^ ^]^ We sought to test whether paternal exposure to HFD could transgenerationally transfer the metabolic phenotypes to the offspring. 4‐week‐old male mice were randomly assigned to either the CD or HFD for 12 weeks. CD‐F0 or HFD‐F0 mice were then mated with 8‐week‐old unexposed female mice to generate the F1, and the same mating scheme was used to generate the F2 (**Figure** **1**A). None of the F1‐F2 offspring were ever directly exposed to HFD. For each generation, the body weight of mice was monitored weekly, starting from 5 weeks until 20 weeks of age. Metabolism phenotypes were monitored at the age of 24 ± 2 weeks.

As shown in Figure S1A0 (Supporting Information), compared to CD‐F0 mice, we found that the weight of HFD‐F0 mice was increased dramatically, but the body weights in female mice of F1 and F2 showed normal body weight, suggesting that postnatal influence can overcome the adverse effect on body weight of paternal obesity (Figure S1A1‐2, Supporting Information). We also found that there was no significant difference in the birth weight of F1 or F2 between the CD and HFD groups (Figure S1E, Supporting Information), whereas the liver weight and liver weight/body weight ratio were significantly increased in the HFD groups (Figure 1E; Figure S1F, Supporting Information), suggesting that paternal HFD exposure may affect the liver development of female offspring. Additionally, paternal HFD exposure led to a slight decrease in litter size of F1 and F2, suggesting that paternal HFD exposure had an adverse influence on male fertility (Figure S1G, Supporting Information). Through metabolism tests, we found that HFD‐F0 mice have higher blood glucose levels in glucose tolerance (Figure S1B0, Supporting Information), insulin sensitivity (Figure S1C0, Supporting Information), and pyruvate tolerance (Figure S1D0, Supporting Information), compared with CD‐F0 mice. Intriguingly, examination of the metabolic phenotypes of F1 and F2 female offspring revealed that HFD mice were unaffected in the blood glucose level of IPPTT, but IPGTT and IPITT blood glucose levels were significantly higher than those in CD offspring (Figure 1B–D, Figure S1B‐D1, Supporting Information), indicating that glucose intolerant and insulin insensitive could be transmitted to the female offspring of F2. Moreover, the serum insulin levels and the homeostasis model assessment of insulin resistance (HOMA‐IR = fasting insulin [µU/mL] × fasting blood glucose [mM]/22.5) in HFD‐F0 and their F1‐F2 female offspring were elevated dramatically (Figure 1F,G), indicating that paternal HFD exposure could induce glucose intolerance and IR, which may be transferred from the F0 mice to the F2 female offspring.

To further determine whether the liver, the key organ closely related to metabolic function, was potentially correlated with those derangements, a series of studies was performed. The liver histological examination of the HFD‐F0 and their F1‐F2 female offspring revealed full‐fat vacuoles in lobule cells, infiltration of inflammatory cells, and cell swelling (Figure 1I). Abnormal glycogen accumulation was examined by PAS staining, as well as triglyceride accumulation evidenced by the Oil Red O staining, suggesting there were structure‐damaged glycogenolysis disorders and impaired lipid clearance in the liver (Figure 1I). Additionally, liver NAS scores and lipid droplet area showed significant increases in the HFD‐F0 group and HFD F1‐F2 female offspring (Figure 1I). To further investigate systemic changes in lipid metabolism, plasma lipid profiles were tested. It was revealed that the TG and LDL‐C levels were significantly increased in the HFD‐F0 group (Figure S1H,I, Supporting Information), and a reduced plasma HDL‐C level was observed (Figure 1H). Notably, the serum HDL‐C levels were kept decreasing in HFD F1‐F2 female offspring (Figure 1H). While serum TG and serum LDL‐C in the F1 and F2 female offspring were no different between CD and HFD groups (Figure S1H,I, Supporting Information). These results suggest systemically impaired lipid metabolism and glycogen accumulation in the female offspring of HFD‐exposed fathers.

To summarize, the assessments of weight, metabolic testing, and histological analysis indicate that paternal HFD exposure could induce transgenerational transmission of glucose intolerant, insulin resistance, impaired fertility, and lower plasma HDL‐C from the F0 mice to the F2 female mice in comparison with CD descendants.

Given that HFD F0 and their F1‐F2 female offspring exhibit enhanced glucose intolerant and IR. To explore the mechanism of metabolic disorder, we evaluated the expression of p‐AKT/AKT and phospho‐glycogen synthase kinase 3 beta (p‐GSK3β)/GSK3β, two important proteins involved in the regulation of glucose metabolism. The phosphorylation of GSK3β was decreased in the liver tissue of HFD‐F0, while the total GSK3β; was unchanged. Thus, the p‐GSK3β;/GSK3β; was significantly decreased in HFD‐F0 (Figure 1J). Further, the p‐AKT protein and p‐AKT/AKT in the liver tissue of HFD‐F0 mice were decreased dominantly compared with CD‐F0 mice. Interestingly, those decreases in p‐AKT/AKT and p‐GSK3β;/GSK3β; were similarly occurring in the liver tissues of HFD‐F1 and HFD‐F2 female mice (Figure 1J).

### 2.2 Paternal Obesity Alters the Liver Transcriptome, Leading to Transgenerational Glucose Metabolic Dysfunction in the Female Offspring

We performed RNA sequencing on the left lateral lobe (lobos hepatics sinister laterals) of adult female mice (F1‐F2) to determine if the altered metabolic state of paternal obesity and their female offspring (F1‐F2) was associated with varying liver gene expression (Figure S2A,B, Supporting Information). We compared hepatic transcription profiles by diet and generation using a gene‐level assessment at single‐transcript resolution. As expected, obesity was correlated with differential hepatic gene expression. Liver from HFD‐F1 female mice showed differential expression of 537 genes in comparing CD‐F1 (Figure S2C, Supporting Information, Lancaster P<0.05). Similarly, when comparing HFD‐F2 to CD‐F2, 151 genes were differentially expressed (Figure S2D, Supporting Information, Lancaster *P*<0.05). Of these differentially expressed genes (DEGs), 29 genes were altered in the liver tissues of both F1 and F2 female offspring of HFD group (Figure S2E, Supporting Information). We looked at the functions of these 29 DEGs using gene ontology (GO) and pathway enrichment analysis. The DEGs were mostly involved in controlling glucose homeostasis, gluconeogenesis, and the endoplasmic reticulum's unfolded protein response (Figure S2F, Supporting Information). Then, we found the *Manf* gene was consistently downregulated in the RNA‐seq data from both F1 and F2 female offspring of HFD groups, which might be associated with the paternal HFD exposure‐induced transgenerational transmitted glucose metabolic dysfunction (Figure S2G, Supporting Information).

### 2.3 MANF Might be Responsible for the Transgenerational Impaired Glucose Metabolic Dysfunction by Inducing ER Stress

**Figure 2:**
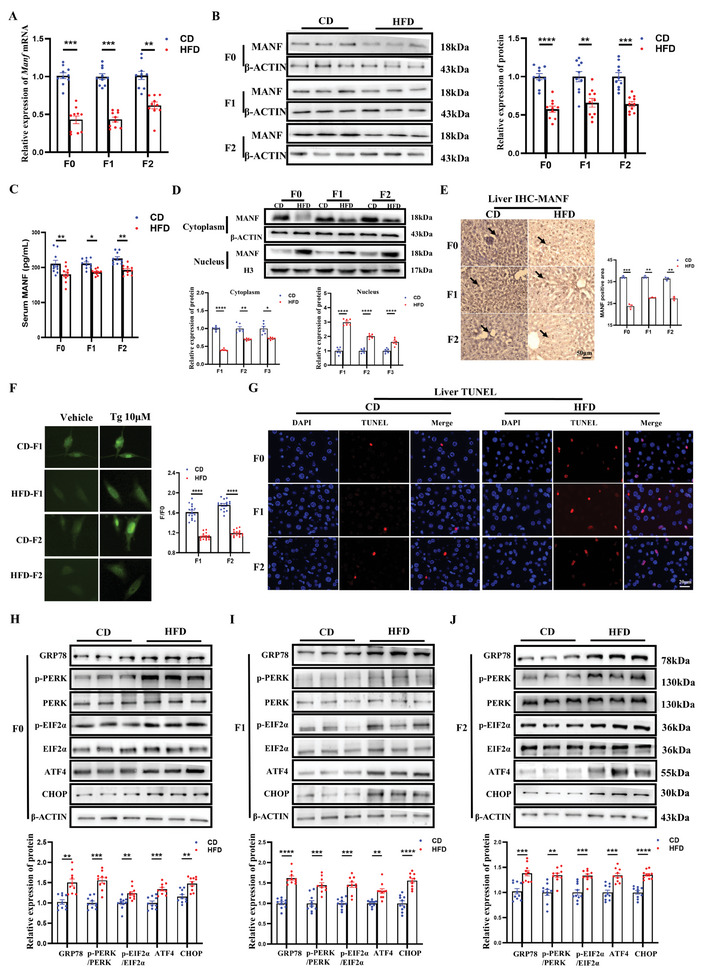
MANF might be responsible for the transgenerational impaired glucose metabolic dysfunction by inducing ER stress. A) The mRNA levels of *Manf* in the liver of F0 male mice and F1–F2 female mice (n = 10 each group); B) Protein levels of MANF in liver from F0 male mice and F1–F2 female mice between CD and HFD groups (n = 10 each group); C) Serum MANF levels of F0 male mice and F1–F2 female mice between CD and HFD groups (n = 10 each group); D) Cytoplasmic and nuclear MANF in liver tissue from F0 male mice and F1–F2 female mice between CD and HFD groups were examined by Western blotting. Cytoplasmic protein β‐actin and nuclear protein histone3 served as controls (n = 6 each group); (E) IHC staining of MANF in liver tissues from F0 male mice and F1–F2 female mice between CD and HFD groups. The black arrows indicate the expression of MANF in nuclear (n = 3 each group); (F) Tg‐mediated calcium content in primary hepatocytes from F1‐F2 female offspring between CD and HFD groups (cell number = 15 per group); G) The TUNEL results in liver tissue from F0 male mice and F1–F2 female mice between the CD and HFD groups (n = 3 each group); (H‐J) Protein levels of GRP78, p‐PERK, PERK, p‐PERK/PERK, p‐EIF2α, EIF2α, p‐EIF2α/EIF2α, ATF4, and CHOP in liver from F0 male mice and F1–F2 female mice between CD and HFD groups (n = 10 each group). Data are mean ± SEM. ns, no significance; ^*^ *p* < 0.05; ^**^ *p* < 0.01; ^***^ *p* < 0.001; ^****^ *p* < 0.0001 (two‐way ANOVA).

MANF, a feeding‐induced hepatokine, acts as a protective mechanism against ER stress‐induced cellular damage to mitigate the progression of diabetes by modulating lipid metabolism, inflammation, apoptosis, and proliferation in the liver, adipose tissue, and pancreatic tissues.^[^ ^22^ ^]^ Using RT‐qPCR and western blotting, we found that the expression of MANF was down‐regulated in HFD groups across all three generations (**Figure** **2**A,B). Since MANF is a secretion protein, we found that the content of MANF in serum was also decreasing (Figure 2C). Notably, MANF could translocate to the nuclei under inflammation or ER stress.^[^ ^23^ ^]^ In the liver tissue of the HFD‐F0 group and their F1‐F2 female offspring, MANF was increased in the nucleus and decreased in the cytoplasm (Figure 2D), which was further validated by IHC of the liver (Figure 2E). To determine whether the impaired glucose metabolic dysfunction in paternal obesity female offspring had ER stress in the liver, we measured ER Ca^2+^ content after Tg (10^−6^ M) incubation in the primary hepatocytes from F1 and F2 female offspring. Obese female offspring had reduced ER Ca^2+^ content levels in F1 and F2 of the HFD group when compared with CD female offspring (Figure 2F), indicating that the glucose metabolic dysfunction in obesity female offspring had ER stress across all three generations. In addition, compared to CD offspring, the apoptosis of liver tissue from HFD‐F0 and their F1‐F2 female offspring was significantly increased in obese offspring by using the TUNEL assay (Figure 2G). Three canonical unfolded protein responses (UPR) signaling pathways, regulated by GRP78, have been delineated: inositol‐requiring protein‐1 (IRE1), activating transcription factor‐6 (ATF6), and protein kinase RNA (PKR)‐like ER kinase (PERK).^[^ ^24^ ^]^ In the current research, we analyzed the expression of genes relevant to the UPR signaling pathways by RT‐qPCR and found that *Grp78* and *Chop* were markedly elevated in the paternal HFD female offspring compared to the CD female offspring (Figure S3A,B, Supporting Information). Thus, we evaluated the protein expression of GRP78 and the PERK‐EIF2α‐ATF4‐CHOP pathway in the liver from the F0 mice and the F1‐F2 female mice of two groups. As shown in Figure 2H,J, the protein levels of p‐PERK/PERK, p‐EIF2α/EIF2α, ATF4, and CHOP were significantly raised in the liver from the HFD‐F0 mice and their F1‐F2 female offspring, suggesting that ER stress and the PERK‐p‐EIF2α‐ATF4‐CHOP pathway may contribute to the glucose metabolic dysfunction upon paternal obesity. Consequently, we hypothesized that MANF, acting as a protective mechanism against ER stress‐induced cellular damage, was the central pathway responsible for the transgenerational transmission of glucose metabolic dysfunction induced by paternal HFD exposure.

**Figure 3:**
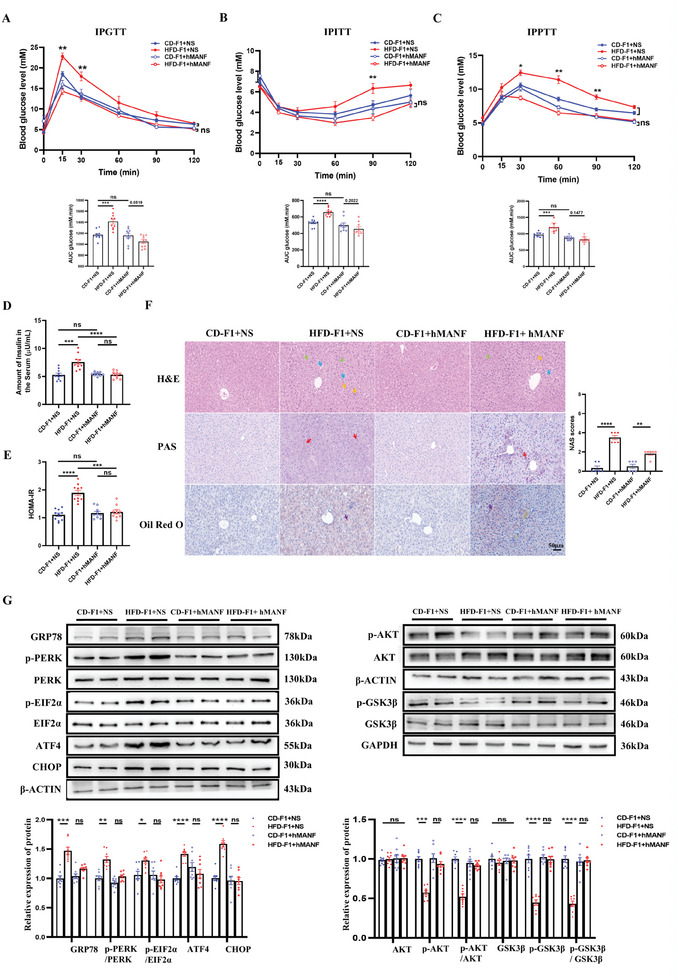
Intravenously injected hMANF in F1 female offspring improves the liver histoarchitecture and reverses glucose metabolic dysfunction and ER stress. At the end of treatment with a recombinant human MANF with a dose of 1.5 ng/kg every 2 days for two weeks by intravenous injections in F1 female offspring. A) IPGTT and AUC (n = 10 each group); B) IPITT and AUC (n = 10 each group); C) IPPTT (n = 10 each group); D) Fasting serum insulin levels (n = 10 each group); E) HOMA‐IR (n = 10 each group); F) The H&E, PAS, and Oil Red O staining (n = 3 each group). Fat vacuoles (blue arrows), infiltration of inflammatory cells (yellow arrows), and cell swelling (green arrows), glycogen accumulation (red arrows), triglyceride accumulation (purple arrows). NAS scores in different groups (n = 6 each group); G) Protein levels of AKT, p‐AKT, p‐AKT/AKT, GSK3β, p‐GSK3β, p‐GSK3β/GSK3β, and the GRP78‐PERK‐EIF2α‐ATF4‐CHOP pathways in liver of different groups (n = 8 each group). Data are mean ± SEM. ns, no significance; ^*^ *p* < 0.05; ^**^ *p* < 0.01; ^***^ *p* < 0.001; ^****^ *p* < 0.0001(one‐way or two‐way ANOVA).

To test the critical role of MANF in the transgenerational impaired glucose metabolic dysfunction, recombinant human MANF (hMANF) at different concentrations was intravenously injected in F1 female offspring as previously described.^[^ ^20^ ^]^ As shown in Figure S4A (Supporting Information), as the administered concentration of hMANF increases, the protein abundance of His‐tag increases. Then, we choose intravenous injection of 1.5 ng kg^−1^ for subsequent experiments. Through glucose metabolism tests, we found that MANF lowered the blood glucose level in HFD‐F1 group and reached the level of the CD‐F1 group at day 14 postinjection in F1 female offspring (**Figure** **3**A–C). Consistent with the normalization of suggesting blood glucose levels in the glucose metabolism tests, MANF also normalized the impaired serum insulin level and HOMA‐IR (Figure 3D,E). However, liver histological examination revealed that after intravenous injection of hMANF in F1 offspring, the number of fat vacuoles, inflammatory cell infiltration, cell swelling, abnormal glycogen accumulation, and triglyceride accumulation in lobular cells were decreased but still existed (Figure 3F). Moreover, the liver NAS scores of hMANF‐treated HFD‐F1 females were lower than those of untreated HFD‐F1 but remained higher than the CD‐F1 group (Figure 3F). These results suggest that the intravenous injection of hMANF in F1 offspring can partly, if not completely, rescue the effects of paternal obesity on liver tissue structure. Following, we evaluated the protein levels of p‐AKT/AKT, p‐GSK3β;/GSK3β;, GRP78, p‐PERK/PERK, p‐EIF2α/EIF2α, ATF4, and CHOP of the liver tissues and then found that MANF normalized the levels of these proteins, further verifying our hypothesis (Figure 3G). These data suggested the essential role of MANF in the obesity‐induced transgenerational transmission of glucose metabolic dysfunction.

**Figure 4:**
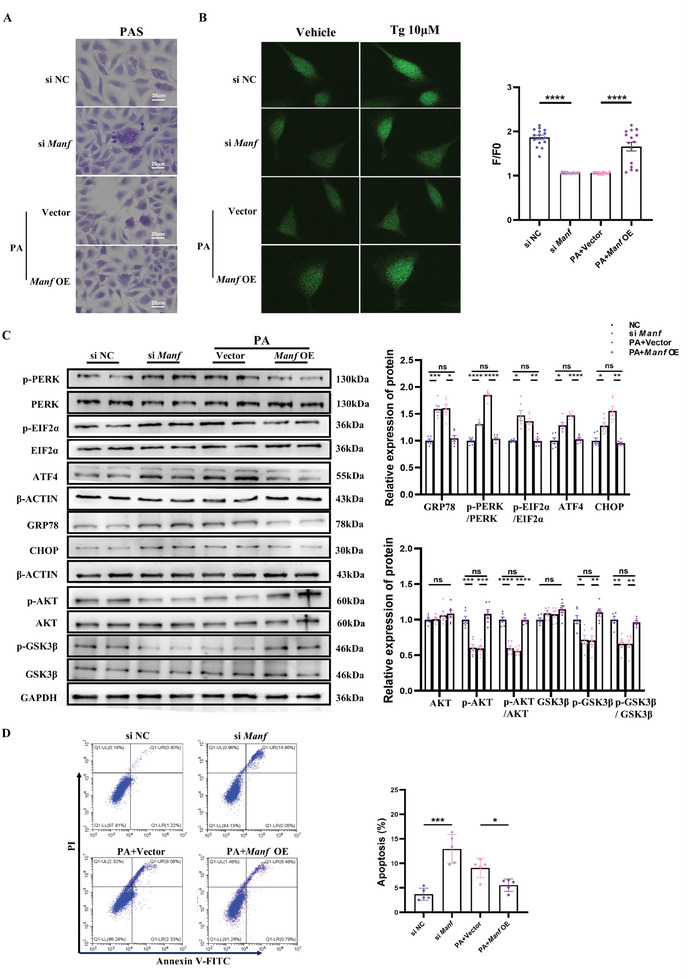
PA reduced the expression of MANF, leading to ER stress, disrupted glucose homeostasis, and apoptosis in vitro. A) PAS staining of primary hepatocytes in different groups (n = 3 each group); B) Tg‐mediated calcium content of primary hepatocytes in different groups (cell number = 15 per group); C) Protein levels of AKT, p‐AKT, p‐AKT/AKT, GSK3β, p‐GSK3β, p‐GSK3β/GSK3β, and the GRP78‐PERK‐EIF2α‐ATF4‐CHOP pathways in primary hepatocytes of different groups (n = 6 each group); D). Apoptotic cells in primary hepatocytes of different groups were identified by using CytoFLEX (n = 6 each group). Data are mean ± SEM. ns, no significance; ^*^ *p* < 0.05; ^**^ *p* < 0.01; ^***^ *p* < 0.001; ^****^ *p* < 0.0001 (two‐tailed t‐test or two‐way ANOVA).

To further investigate the association between MANF, ER stress, and glucose metabolic dysfunction, primary hepatocytes were transfected with *Manf* siRNA (s*i Manf*) or *Manf* overexpression plasmids (*Manf* OE). We isolated primary hepatocytes from 4‐week‐old female mice and identified them by PAS and cytokeratin 18 (CK18) (Figure S4B, Supporting Information). Initially, we assessed the efficiency of gene knockdown. As shown in Figure S4C (Supporting Information), the mRNA and protein levels of MANF were significantly reduced when the primary hepatocytes were transfected with *Manf* siRNA. Secondly, we determined the effect of the concentration gradient of PA on the expression of *Manf* and found that the mRNA expression of *Manf* was decreased with the increase of PA concentration (Figure S4D, Supporting Information). Then, we selected 0.2 mM PA to verify the effect of time gradient on MANF expression and found that the expression of MANF was increased in the 12 h but was decreased in 24 and 48 h (Figure S4E, Supporting Information). These results suggest that MANF was adaptively increased in the early stage of high‐fat stimulation, while MANF expression decreases during chronic high‐fat stimulation. Therefore, we chose 0.2 mMm PA for 48 h for subsequent in vitro experiments. After being included in PA for 48 h, primary hepatocytes were transfected with *Manf* overexpression plasmids for 48 h. And then we found that knockdown of *Manf* and inclusion of PA substantially increase abnormal glycogen accumulated in the primary hepatocytes, whereas increased expression of MANF could rescue this phenomenon (**Figure** **4**A). We also found that the Ca^2+^ content of ER was significantly decreased in *siManf* and PA groups, while the Ca^2+^ content of ER will return to normal levels after transfection with *Manf* overexpression plasmids (Figure 4B). Meanwhile, knockdown of *Manf* and inclusion of PA would decrease the protein level of p‐AKT/AKT and p‐GSK3β;/GSK3β;, but substantially increase the protein level of GRP78, p‐PERK/PERK, p‐EIF2α/EIF2α, ATF4, and CHOP. Moreover, MANF overexpression can rescue these changes of the above proteins to normal levels (Figure 4C). We also evaluated the apoptotic activity in primary hepatocytes of different groups by using CytoFLEX, then found that knockdown of *Manf* and inclusion of PA could accelerate the apoptosis of primary hepatocytes (Figure 4D). These results indicated that the reduction of MANF would induce ER stress, apoptosis, and an increase in abnormal glycogen accumulated in the primary hepatocytes.

### 2.4 EZH2 Increases H3K27me3 Modification, Leading to Transgenerational Deregulation of MANF in Obese Offspring

**Figure 5:**
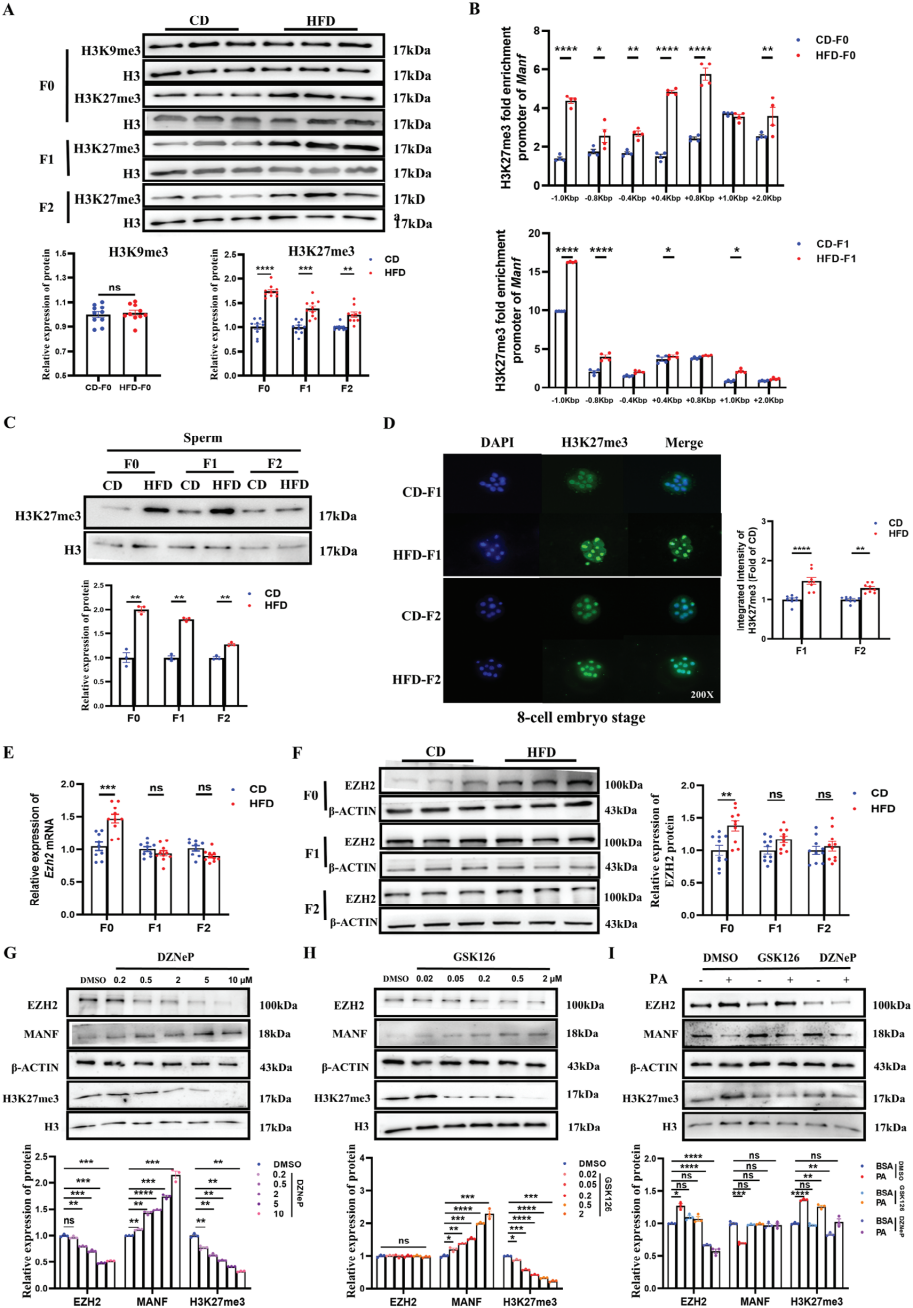
Paternal obesity enhanced EZH2‐H3K27me3 modification, leading to transgenerational deregulation of the *Manf* gene in offspring. A) The protein levels of H3K9me3 and H3K27me3 in the liver of F0 male mice, as well as the protein levels of H3K27me3 in the liver of F1 and F2 female mice (n = 10 each group); B) ChIP‐RT‐qPCR detecting H3K27me3 binding to the promoter region of the *Manf* gene in liver tissue of F0 male mice and F1 female mice (n = 4 each group). Normalized to Input; C) The protein levels of H3K27me3 in the sperm of F0‐F2 male mice (n = 6 each group); D) The immunofluorescent staining of H3K27me3 (green) in 8‐cell embryo stage from F1‐F2 generation between CD and HFD groups (n = 8 each group). Nuclei are labeled by DAPI (blue). 200X. E) The mRNA levels of *Ezh2* in the liver of F0 male mice and F1–F2 female mice (n = 10 each group); F) Protein levels of EZH2 in liver from F0 male mice and F1–F2 female mice between CD and HFD groups (n = 10 each group); (G) The protein levels of EZH2, H3K27me3, and MANF were measured in primary hepatocytes following treatment with a concentration gradient of DZNep (n = 3 each group); H) The protein levels of EZH2, H3K27me3, and MANF were measured in primary hepatocytes following treatment with a concentration gradient of GSK126 (n = 3 each group); I) The protein levels of EZH2, H3K27me3, and MANF in primary hepatocytes following treatment with or without PA, GSK126, or DZNep (n = 3 each group). Data are mean ± SEM. ns, no significance; ^*^ *p* < 0.05; ^**^ *p* < 0.01; ^***^ *p* < 0.001; ^****^ *p* < 0.0001 (two‐tailed t‐test or two‐way ANOVA).

It is well known that both fertility and paternally transmitted effects have been reported to be associated with epigenomic modification, including DNA methylation, non‐coding RNA, and histones.^[^ ^5^, ^8^, ^9^, ^25^ ^]^ To further investigate the mechanism of the transgenerational transmitted downregulation of the *Manf* gene, we initially investigated epigenomic modifications that may drive the transcriptional activation of the *Manf* gene. A major epigenetic modification is histone modification, which controls the looseness and density of chromatin to regulate gene accessibility and cause heritable changes in target gene transcription.^[^ ^26^ ^]^ Since *Manf* was downregulated in obese offspring, we investigated two major histone modifications, which are associated with gene repression,^[^ ^27^ ^]^ in the livers from CD‐F0 and HFD‐F0 groups and found that H3K27me3 was increased significantly in the HFD‐F0 group, whereas H3 lysine 9 trimethylation (H3K9me3) remained unchanged (**Figure** **5**A). Thus, we concentrated on studying H3K27me3. After that, we compared the H3K27me3 expression in the livers of F1 and F2 female offspring and discovered that, in contrast to CD female offspring, the obese female offspring had a persistently higher level of H3K27me3 expression (Figure 5A). By H3K27me3 ChIP‐qPCR analysis, we found that the H3K27me3 binding on the *Manf* promoter was increased in the liver tissue of HFD‐F0 and HFD‐F1 female offspring (Figure 5B). According to these findings, it was suggested that the *Manf* gene promoters increased deposition of H3K27me3 and strengthened its gene‐repressive effect and had a role in the persistent downregulation of MANF in obese female offspring. Considering that the increase of H3K27me3 may have originated from HFD and been transmitted through the sperm of HFD‐exposed male mice, we investigated the protein levels of H3K27me3 in the sperm of the F0‐F2 with or without HFD exposure. We found that, compared to the CD group, the protein level of H3K27me3 was significantly upregulated in the sperm of the HFD F0‐F2 male mice (Figure 5C). The increase in H3K27me3 level was also observed in the 8‐cell embryo stage in vitro through immunofluorescence of HFD F1‐F2 than in CD F1‐F2 (Figure 5D), indicating that the elevated H3K27me3, induced by HFD exposure, will be transmitted across generations.

Since PRC2, comprised of embryonic ectoderm development (EED), enhancer of zeste 1/2 (EZH1/2), and suppressor of zeste 12 (SUZ12), mediates H3K27me3 to maintain transcriptional silencing,^[^ ^28^ ^]^ we determined the mRNA expression levels of PRC2's components in the liver with or without HFD exposure. We found that, compared to the CD‐F0 group, the *Ezh2* mRNA expression was upregulated in the HFD‐F0 group, while there was no change in the mRNA expression of *Eed*, *Ezh1*, or *Suz12* (Figure S5A, Supporting Information). In agreement with the aforementioned results, the protein level of EZH2 was enhanced significantly in the HFD‐F0 group than in the CD‐F0 group (Figure 5E,F). We, therefore, focused on EZH2. Intriguingly, the increased expression of EZH2 was only observed in the liver tissue of F0 but not in F1 or F2 female offspring (Figure 5E,F), indicating that H3K27me3 is initially enhanced by EZH2 but subsequently inherited. Additionally, we assessed the relationship among EZH2, H3K27me3, and MANF by examining whether EZH2 directly regulates H3K27me3 levels to reduce MANF expression. A concentration gradient of DZNep (an inhibitor of all S‐adenosylmethionine [SAM] dependent enzymes, including EZH2)^[^ ^29^ ^]^ and GSK126 (a selective inhibitor of EZH2 methyltransferase activity)^[^ ^30^ ^]^ was utilized to reprogram the epigenetic pathways in primary hepatocytes. GSK126 significantly inhibited H3K27me3 levels without altering EZH2 expression, whereas DZNep concurrently decreased EZH2 expression (Figure 5G,H). Interestingly, both GSK126 and DZNep significantly reduced the level of H3K27me3 while increasing MANF expression (Figure 5G,H). Inhibiting EZH2 through GSK126 or DZNep treatment effectively enhanced PA‐induced MANF expression while decreasing H3K27me3 expression in primary hepatocytes (Figure 5I). Nevertheless, the knockdown of *Manf* did not influence the expression of H3K27me3 and EZH2 (Figure S5B, Supporting Information). Meanwhile, overexpression of *Manf* in primary hepatocytes after PA treatment didn't affect the expression of EZH2 and H3K27me3 (Figure S5B, Supporting Information). These findings indicate that H3K27me3 modification at the promoter level is involved in EZH2‐mediated MANF repression.

According to reports, EZH2 may also act as a platform for the recruitment of the DNA methyltransferase DNMT1.^[^ ^31^ ^]^ We used Co‐IP with specific DNMT1 and EZH2 antibodies to investigate the physical interaction between DNMT1 and EZH2. Co‐IP studies showed a mutual integration and a notable increase in EZH2 and DNMT1 in HFD‐F0 mice's liver tissue, which was consistent with the increase in DNMT1 in the mice's serum (Figure S6A,B, Supporting Information). Further, to relate the relationship between DNMT1, EZH2, H3K27me3, and MANF, primary hepatocytes were applied to a concentration gradient of DC‐05 (a selective inhibitor of DNMT1).^[^ ^32^ ^]^ As shown in Figure S6C (Supporting Information), DC‐05 reduced the level of H3K27me3 without affecting EZH2 expression while increasing MANF expression in primary hepatocytes, suggesting that the expression of H3K27me3 and MANF is regulated by DNMT1. However, the result of bisulfite sequencing showed that compared to CD‐F0, there was no difference in the methylation level of *Manf* in the liver of HFD‐F0 mice, and barely methylated (Figure S6D, Supporting Information). Meanwhile, the methylation level of *Manf* in the liver between CD‐F1 and HFD‐F1 female mice was also no different (Figure S6E, Supporting Information), indicating that DNA methylation modification might not be involved in the transgenerational regulation of *Manf* in paternal obese female offspring.

**Figure 6:**
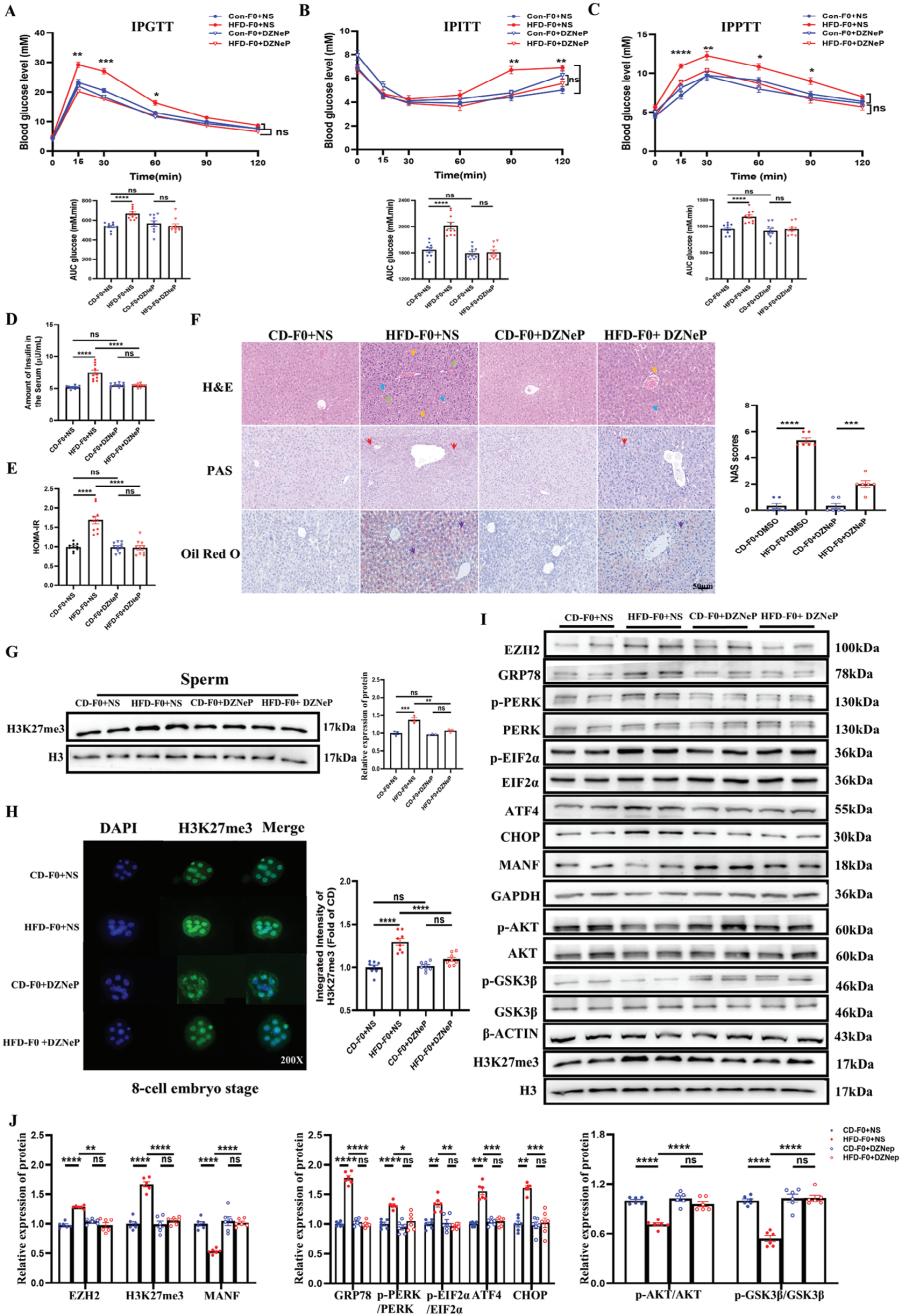
Intraperitoneally injected DZNep in F0 male mice improves the liver histoarchitecture and reverses glucose metabolic dysfunction and ER stress, with altering H3K27me3 in the sperm and 8‐cell embryo stage. At the end of treatment with a DZNep with a dose of 1 mg kg^−1^ every 2 days for two weeks through intraperitoneal injection in F0 male mice. (A) IPGTT and AUC (n = 10 each group); B) IPITT and AUC (n = 10 each group); C) IPPTT (n = 10 each group); D) Fasting serum insulin levels (n = 10 each group); E) HOMA‐IR (n = 10 each group); F) The H&E, PAS, and Oil Red O staining (n = 3 each group). Fat vacuoles (blue arrows), infiltration of inflammatory cells (yellow arrows), and cell swelling (green arrows), glycogen accumulation (red arrows), triglyceride accumulation (purple arrows). NAS scores in different groups (n = 6 each group); G) The protein levels of H3K27me3 in the sperm of F0 male mice with or without treatment of DZNep (n = 6 each group); H) The immunofluorescent staining of H3K27me3 (green) in 8‐cell embryo stage of different groups (n = 8 each group). Nuclei are labeled by DAPI (blue). 200X; I,J) Protein levels of the EZH2‐H3K27me3‐MANF pathway, AKT, p‐AKT, p‐AKT/AKT, GSK3β, p‐GSK3β, p‐GSK3β/GSK3β, and the GRP78‐PERK‐EIF2α‐ATF4‐CHOP pathways in liver of different groups (n = 6 each group). Data are mean ± SEM. ns, no significance; ^*^ *p* < 0.05; ^**^ *p* < 0.01; ^***^ *p* < 0.001; ^****^ *p* < 0.0001 (one‐way or two‐way ANOVA).

To demonstrate the regulatory role of EZH2 on H3K27me3 and MANF in mice, DZNep (1.5 ng kg^−1^) was intraperitoneally injected in the F0 group at 16 weeks for 2 weeks. Through glucose metabolism tests (IPGTT, IPITT, and IPPTT), we found that a downregulation of EZH2 could lower the blood glucose level in the HFD‐F0 group and reach the level of the CD‐F0 group (**Figure** **6**A–C). Meanwhile, the level of insulin in HFD‐F0 serum and HOMA‐IR also normalized (Figure 6D,E). Histological analysis of F0 liver revealed that DZNep treatment partially reversed HFD‐induced structural damage (Figure 6F). Consistently, the liver NAS scores of DZNep‐treated HFD‐F0 mice were significantly lower than those of untreated HFD‐F0 mice but remained elevated relative to the CD‐F0 group (Figure 6F). We also determined the protein expression of H3K27me3 in sperm and 8‐cell embryo stages in F0 groups and found that they reached the level of the CD‐F0 group after DZNep treatment in HFD‐F0 mice (Figure 6G,H). Then, we evaluated the protein levels of EZH2, H3K27me3, MANF, p‐AKT/AKT, p‐GSK3β/GSK3β, GRP78, p‐PERK/PERK, p‐EIF2α/EIF2α, ATF4, and CHOP of the liver tissues and then found that the downregulated expression of EZH2 by DZNep could normalize the levels of these proteins (Figure 6I). These data suggested that EZH2, which regulates the expression of H3K27me3 and MANF, plays a critical role in the obesity‐induced transgenerational transmission of glucose metabolic dysfunction.

### 2.5 PA Increased the EZH2‐H3K27me3‐MANF Pathway, Leading to ER Stress and the Accumulation of Abnormal Glycogen

**Figure 7:**
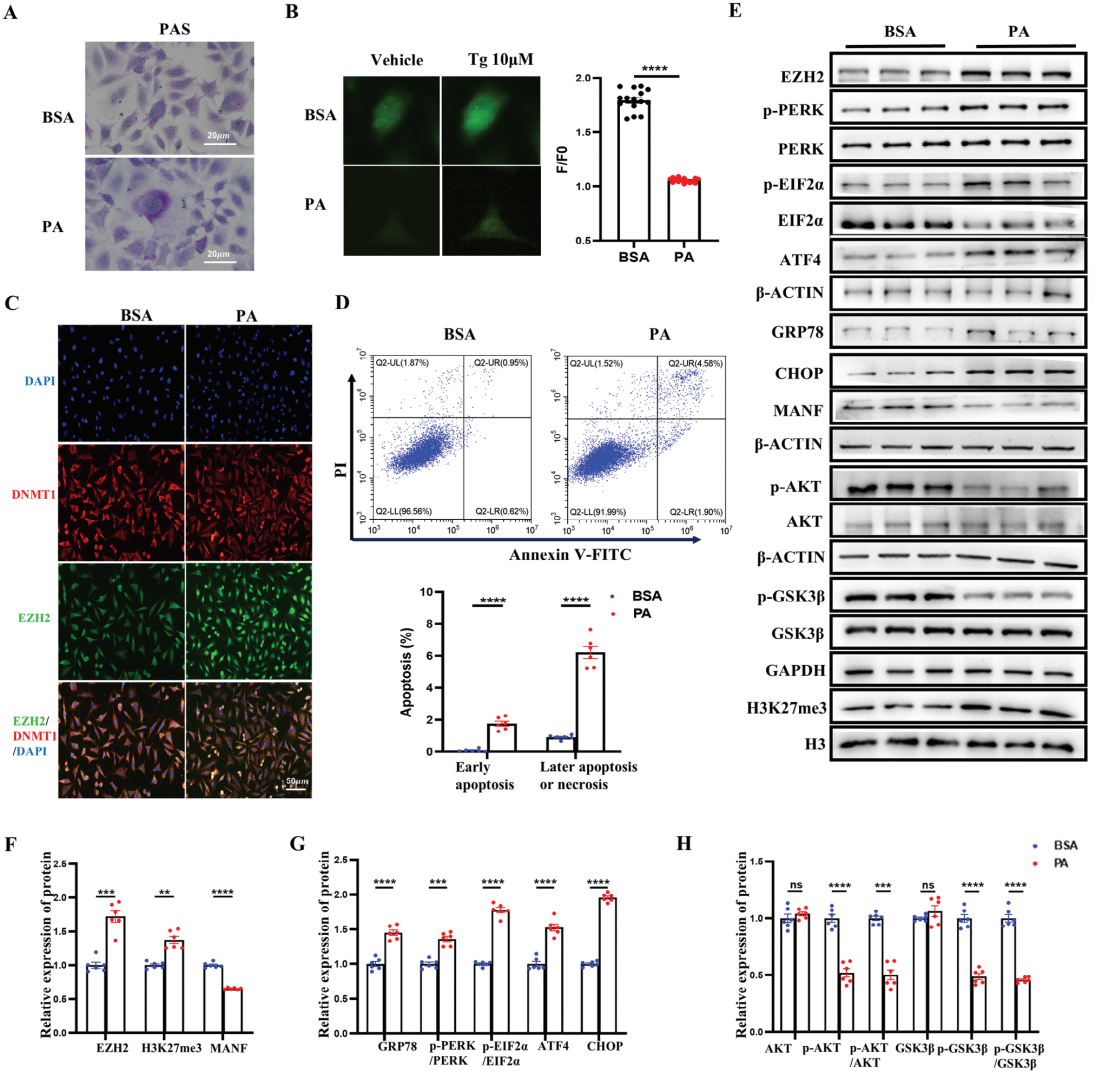
PA enhanced the activation of the EZH2‐H3K27me3‐MANF pathway, leading to ER stress, glucose homeostasis dysfunction and apoptosis in vitro. A) PAS staining of primary hepatocytes in different groups (n = 3 each group); B) Tg‐mediated calcium content of primary hepatocytes in different groups (cell number = 15 per group); C) Representative immunofluorescent staining of EZH2 (green) and DNMT1 (red) in primary hepatocytes of different groups (n = 3 each group). Nuclei are labeled by DAPI (blue). Scale bar: 50 µm; D) Apoptotic cells in primary hepatocytes of different groups were identified by using CytoFLEX (n = 6 each group); E) Representative WB of the EZH2‐H3K27me3‐MANF pathway, AKT, p‐AKT, p‐AKT/AKT, GSK3β, p‐GSK3β, p‐GSK3β/GSK3β, and the GRP78‐PERK‐EIF2α‐ATF4‐CHOP pathways in primary hepatocytes of different groups; F) Protein levels of the EZH2‐H3K27me3‐MANF pathway in primary hepatocytes of different groups (n = 6 each group); G) Protein levels of the GRP78‐PERK‐EIF2α‐ATF4‐CHOP pathways in primary hepatocytes of different groups (n = 6 each group); H) Protein levels of AKT, p‐AKT, p‐AKT/AKT, GSK3β, p‐GSK3β, p‐GSK3β/GSK3β in primary hepatocytes of different groups (n = 6 each group); Data are mean ± SEM. ns, no significance; ^*^ *p* < 0.05; ^**^ *p* < 0.01; ^***^ *p* < 0.001; ^****^ *p* < 0.0001 (two‐tailed t‐test or two‐way ANOVA).

According to this study, exposure to HFD before mating led to activation of the PERK‐EIF2α‐ATF4‐CHOP pathway and decreased p‐AKT/AKT and p‐GSK3β;/GSK3β;, which was due to ER stress caused by the enhanced EZH2‐H3K27me3‐MANF pathway, ultimately leading to impaired transgenerational glucose metabolic dysfunction in the liver. To determine whether ER stress and glucose metabolic dysfunction are caused by HFD stimulation, primary hepatocytes were incubated by PA, used to simulate the effects of high‐fat exposure on primary hepatocytes in vitro. The results of PAS staining showed that glycogen accumulation in the PA group was noticeably greater than that in the BSA group (**Figure** **7**A). As shown in Figure 7B, the ER Ca^2+^ concentration was lower in the PA group than the BSA group. Meanwhile, after incubation of PA, the expression of EZH2 was elevated significantly and transferred from the cytoplasm to the nucleus (Figure 7C). The early and later apoptosis levels of the two groups, measured by an annexin V‐FITC/PI kit, were significantly increased in the PA group compared with the BSA group (Figure 7D). We also found that compared with the BSA group, the protein levels of p‐AKT/AKT, p‐GSK3β;/GSK3β;, and MANF were decreased, while the protein levels of EZH2, H3K27me3, GRP78, p‐PERK/PERK, p‐EIF2α/EIF2α, ATF4, and CHOP were increased in the PA group (Figure 7E–H). In conclusion, PA incubation for 48 h induced ER stress, abnormal glycogen deposition, and apoptosis in primary hepatocytes.

To gain more insight into the relationship of ER stress and the EZH2‐H3K27me2‐MANF pathway, primary hepatocytes were incubated by DMSO or Tg (10^−6^ m), an ER stress inducer, for 48 h. Firstly, we determined the efficiency of Tg in inducing ER stress by elevating the PERK‐EIF2α ‐MANF pathway. As shown in Figure S7A (Supporting Information), compared to the DMSO group, the protein levels of GRP78, p‐PERK/PERK, p‐EIF2α/EIF2α, ATF4, and CHOP were increased in the Tg group. PAS staining showed that glycogen was abnormally accumulated in the Tg group compared to that in the DMSO group (Figure S7B, Supporting Information). We also determined the protein expression of p‐AKT/AKT and p‐GSK3β;/GSK3β; and found they were decreased in the Tg group (Figure S7C, Supporting Information). Meanwhile, under the influence of ER stress, the protein levels of MANF were significantly decreased in the Tg group compared with the DMSO group. While the expression of EZH2 and H3K27me3 showed no difference between the two groups (Figure S7D, Supporting Information). These results indicated that ER stress could induce abnormal glycogen deposition and the downregulation of MANF expression but has no effect on the expression of EZH2 and H3K27me3. The above results showed that ER stress was a direct result of HFD exposure.

## 3 Discussion

The prevalence of diabetes and obesity has drastically increased over the past few decades. Many clinical and animal model studies indicate that diabetes is a multifactorial disease with substantial genetic risk.^[^ ^33^ ^]^ However, numerous large‐scale genetic studies have found that genetic variants accounted for only a small proportion of variation in diabetes, suggesting that epigenetic regulation mechanisms may be involved in the heritability of diabetes.^[^ ^34^ ^]^ Paternal diet influences offspring metabolism through intergenerational transmission of epigenetic modifications (e.g., DNA methylation, histone markers, ncRNA).^[^ ^35^ ^]^ However, the underlying mechanism is still not clear.

Our study established a paternal HFD exposure model to investigate the role of epigenetic mechanisms in the transgenerational inheritance of glucose metabolic dysfunction. After HFD exposure for 12 weeks, the HFD‐F0 group exhibited significantly higher body weight compared to the CD‐F0 group. Although there was no difference in body weight between the F1 and F2 female offspring of the HFD‐F0 and CD‐F0 groups, the liver weight and liver/body weight ratio significantly increased in the HFD groups, indicating that paternal exposure to HFD could impair liver development. Our previous studies have found that paternal HFD exposure increased hepatic gluconeogenesis in male offspring without significantly affecting their glucose tolerance and insulin sensitivity.^[^ ^21^ ^]^ While our present study found that the results of metabolic testing were different in female offspring of paternal HFD exposure, which showed decreased glucose tolerance and insulin sensitivity but were almost unaffected in hepatic gluconeogenesis of F1‐F2 compared with CD female offspring, indicating the sex‐specific effect in offspring. However, it is unclear how HFD exposure in F0 males affects the metabolism of F1‐F2 female offspring and why such transgenerational effects are sex‐specific, which will be the focus of our further research. Thus, we focused on elucidating the molecular mechanism of transgenerational inheritance of glucose metabolic dysfunction in female offspring caused by paternal HFD exposure in the present study.

To search for candidate genes and pathways responsible for the transgenerational glucose metabolic dysfunction, we detected the alteration of the transcriptome in the liver of F1 and F2 female offspring using RNA‐seq analysis. Among the commonly DEGs between the CD group and the HFD group in the F1 and F2 female offspring, MANF was significantly reduced in HFD offspring compared to CD offspring. MANF is an evolutionarily conserved regulator of systemic, especially liver, metabolic homeostasis.^[^ ^19^ ^]^ The increasing metabolic demands, caused by HFD exposure, on the ER, a major site of protein folding and lipid synthesis, lead to ER stress and UPR, which is strongly linked to the emergence of insulin resistance.^[^ ^36^ ^]^ In the absence of ER stress, MANF is retained in the ER by the calcium‐dependent interaction with GRP78, which helps in maintaining the UPR sensors. While depletion of ER calcium levels triggers the dissociation of MANF from the MANF‐GRP78 complex during ER stress, thereby facilitating its subsequent secretion.^[^ ^37^ ^]^ In the present study, the primary hepatocytes of obese female offspring had decreased ER Ca^2+^ content levels in F1 and F2 mice than in CD mice, while the serum MANF levels in HFD‐F0 mice and the F1‐F2 female mice of paternal obesity were lower than in CD mice. Therefore, MANF can potentially serve as a diagnostic biomarker for glucose metabolic dysfunction. The protein expression of GRP78‐PERK‐EIF2α‐ATF4‐CHOP, the UPR sensors, was significantly higher in obesity F0 and their F1‐F2 female mice than CD mice. Meanwhile, the protein expression of the p‐AKT/AKT ratio and p‐GSK3β/GSK3β, markers of glucose metabolic dysfunction, was decreased in the F0 and F1‐F2 female mice of the HFD group compared to the CD group. In HFD‐exposed female offspring, the delivery of recombinant hMANF rescued their glucose metabolic dysfunction and changes in liver structure. Treatment of hMANF also rescued the protein levels of the GRP78‐PERK‐EIF2α‐ATF4‐CHOP pathway and the ratio of p‐AKT/AKT and p‐GSK3β/GSK3β, which are important in the progression of glucose metabolic dysfunction. Our finding further emphasizes the critical role of MANF, a promising therapeutic candidate for diabetes, in the transgenerational inheritance of glucose metabolic dysfunction.

Epigenetic modifications, including histone modification, DNA methylation, small non‐coding RNAs, etc., have gained extensive attention in the transgenerational inheritance of paternal programmed diseases.^[^ ^8^, ^38^ ^]^ We assessed the DNA methylation level, which is a potent inheritable epigenetic factor inhibiting the expression of specific genes. Amazingly, however, there was no difference in DNA methylation levels in the promoter regions of *Manf* between the CD and HFD groups in F0 mice and F1 female mice, indicating that DNA methylation modification might not be involved in the transgenerational regulation of *Manf* in paternal obesity female offspring. After screening, we focused on H3K27me3, a histone modification that consistently increased in the offspring of paternal obesity. ChIP‐qPCR confirmed that elevated H3K27me3 occupancy at the *Manf* promoter correlates with its transgenerational downregulation. Furthermore, we found that the H3K27me3 levels in the HFD group were significantly higher than those in the CD group in the sperm of the F0 and F1 male mice and in the 8‐cell embryo stage of the F1 and F2 generations. These findings provided more support for the theory that heredity entails the germline's epigenetic modifications transferring the effects of paternal exposure to the progeny.

Moreover, EZH2, a component of PRC2, was detected in F0 and their F1‐F2 female offspring, but EZH2 was only significantly increased in HFD‐F0 mice compared with CD mice, indicating that the upregulation of EZH2 is a direct result of HFD exposure. By successfully injecting DZNep intraperitoneally in F0 mice and addressing questions at in vivo, cellular, and molecular levels, we provide an exhaustive approach for understanding the mechanisms linking the EZH2‐H3K27me3‐MANF pathway to programmed glucose metabolic dysfunction in generations following paternal obesity in this study. Intraperitoneal injection of DZNep, an inhibitor of EZH2, in the F0 group successfully improved the impaired glucose metabolic dysfunction, with recovery of the upregulation of EZH2, amelioration of the increased H3K27me3 level, and increased MANF expression in the liver of HFD‐F0 mice. Intriguingly, we also found that DZNep treatment of F0 mice was sufficient to rescue sperm H3K27me3 levels in HFD‐F0 males and 8‐cell embryo stages of the HFD‐F1 group, suggesting that HFD exposure may serve as the trigger that drives the EZH2‐regulated H3K27me3. Once H3K27me3 is carried over and evades offspring embryonic reprogramming, the epigenetic cascade may evade embryonic reprogramming and perpetuate across generations. In conclusion, our study suggests that DZNep may be a potential therapeutic strategy to prevent transgenerational glucose metabolism dysfunction caused by paternal obesity.

Notably, this study has several limitations. Although we demonstrated that the MANF‐PERK‐EIF2α‐ATF4‐CHOP pathway in the liver was essential for the development of transgenerationally transmitted glucose metabolic dysfunction and apoptosis, there are probably other pathways, together with the MANF‐PERK‐EIF2α‐ATF4‐CHOP axis, that affect the blood glucose levels, which need to be further determined. Moreover, the present study didn't validate MANF expression in other somatic tissues, such as the kidneys, pancreas, and muscles. Therefore, MANF may be involved in the function of other organs, leading to glucose metabolism dysfunction together with its function in the liver system. Furthermore, we didn't perform RNA‐seq studies in other blood glucose‐regulating organs, such as the kidney, pancreas, brain, and muscle. Thus, there may be other mechanisms to get along with the liver system, responsible for the susceptibility of glucose metabolic dysfunction across generations. In addition, we screened out and validated the role of EZH2‐H3K27me3 on the regulation of the *Manf* gene, which was transgenerationally decreased in obese offspring. However, EZH2, a component of the PRC2 complex, not only regulated H3K27me3 but also other epigenomic modifications. H3K27me3 may influence multiple gene loci, we don't know whether or how other genes regulated by EZH2‐H3K27me3 are involved in the regulation of blood glucose. What's more, although we identified the up‐regulation of H3K27me3 in the sperm and 8‐cell embryo stage of the obesity group, it's unclear how the histone modification H3K27me3 escapes reprogramming during early embryonic development. Further studies are necessary to explore more fully the mechanisms for the transgenerationally transmitted glucose metabolic dysfunction and apoptosis of liver tissue.

**Figure 8:**
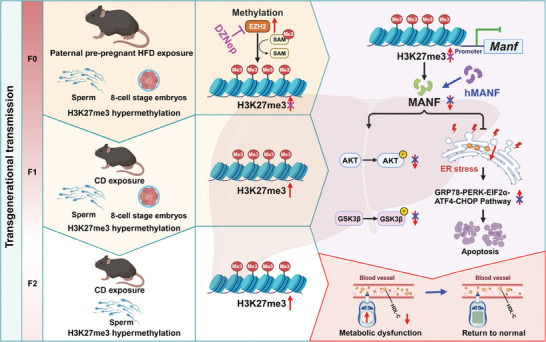
The working model for paternal obesity induces transgenerational metabolic dysfunction in the female offspring via the H3K27me3‐MANF pathway. Induced by paternal pre‐pregnancy HFD exposure, the elevation of H3K27me3 in the sperm passes down from the F0 male mice to the F2 female offspring, is the cause of the transgenerational decrease of *Manf*, leading to ER stress and activation of the GRP78‐PERK‐EIF2α‐ATF4‐CHOP axis and thus resulting in problems with glucose metabolism and apoptosis in the liver of female offspring. However, these get better with each generation (Created with BioRender.com).

Taken together, the present study showed that paternal HFD exposure, resulting in obesity in the HFD‐F0 group, can induce the transgenerational transmission of glucose metabolic dysfunction and apoptosis in F1‐F2 female offspring. Meanwhile, paternal HFD exposure upregulates EZH2, elevates H3K27me3 levels, and decreases the expression of MANF in the liver of F0 offspring, which induces ER stress, leading to the activation of the PERK‐EIF2α‐ATF4‐CHOP axis, resulting in glucose homeostasis dysfunction and apoptosis. Additionally, paternal HFD exposure caused an increased level of H3K27me3 in F0 and F1 sperm and their 8‐cell stage embryos, resulting in transgenerationally transmitted glucose metabolic dysfunction and apoptosis (**Figure** **8**, created using the BioRender platform, accessible at https://BioRender.com). Our study links EZH2‐H3K27me3 with the decrease of MANF and provides a direction for finding effective therapeutic strategies for treating the paternal obesity‐induced transgenerational glucose metabolic dysfunction.

## 4 Experimental Section

### Animals

C57BL/6J mice were used throughout the study and housed five per cage in a temperature‐controlled room (22 ± 2 °C) with a 12 h light/dark cycle and free access to food and water. All procedures relating to animal care and treatment were performed according to the guidelines of the Animal Resource Committee of Soochow University. Male mice (age 4 weeks, 10.2–11.8 g) were randomly assigned to either a control diet (CD; 10% kcal fat) or an HFD (42% kcal fat) for 12 weeks. Their body weight was monitored weekly, followed by a metabolic test. After HFD exposure, 10 mice of each group were mated with 8‐week‐old normal females in a ratio of 1:2 to produce the F1 offspring, respectively. For 3‐Deazaneplanocin A (DZNep) experiments, the remaining male mice in the CD‐F0 or HFD‐F0 group were treated with an EZH2 inhibitor at a dose of 1 mg kg^−1^ every 2 days for two weeks through intraperitoneal injection (N = 10).^[^ ^39^ ^]^ At the age of 16 weeks, 10 F1 male mice from each group were mated with 8‐week‐old normal females to produce the F2 offspring. Litter sizes (number of pups per litter) were recorded, and the birth weight of one pup, randomly selected from each litter, was calculated, respectively. The F1‐F2 generation mice were weaned at 21 days after giving birth, maintained on an ad libitum CD throughout life, and their body weights were monitored weekly until 20 weeks of age. At 24 ± 2 weeks, 10 mice, randomly chosen from each litter, were selected from F1 and F2 female offspring for metabolic testing and then they were sacrificed to collect serum and liver tissue for further analyses. Meanwhile, 10 F1 female mice, randomly selected from each group, were treated with a recombinant human MANF (CX02, Novoprotein, China) at a dose of 1.5 ng kg^−1^ every 2 days for two weeks by intravenous injections at the age of 22 weeks.^[^ ^20^, ^40^ ^]^ At the end of different treatments, mice were subjected to metabolic tests and then followed a fasting period of 12 h before being sacrificed to collect serum, liver tissue, and/or sperm samples for subsequent experimental assays.

### Metabolic Testing

For the intraperitoneal glucose tolerance test (IPGTT) and the intraperitoneal pyruvate tolerance test (IPPTT), mice were fasted overnight (16 h) before receiving an intraperitoneal injection of glucose and pyruvate at the indicated dosage (1.5 g kg^−1^).^[^ ^20^, ^21^, ^41^ ^]^ For the intraperitoneal insulin tolerance test (IPITT), mice were fasted for 4 h before receiving an intraperitoneal injection of 1 IU insulin/kg (P3376, Beyotime, China). Blood glucose levels were measured by using the blood glucose meter (OneTouch, Johnson & Johnson) at baseline (0), 15, 30, 60, 90, and 120 min after glucose injection.

### Histological Analysis

For hematoxylin‐eosin (H&E) and periodic acid‐schiff (PAS) staining, liver tissues were fixed in 4% paraformaldehyde, dehydrated, embedded in paraffin, sectioned at 5 µm, and stained with H&E or PAS. For immunohistochemistry (IHC), sections of tissues were deparaffinized, rehydrated, and underwent antigen retrieval by using heat‐induced epitope methods to measure the expression of MANF according to the manufacturer's instructions (PK10017, Proteintech, China). Frozen liver sections (8 µm) were used for Oil Red O staining. The images were obtained by using a Leica TCS SP8 confocal microscope. The images were captured by a light microscope (Nikon, Japan). The nonalcoholic fatty liver disease (NAFLD) activity score (NAS) system described by Kleiner et al.^[^ ^42^ ^]^ was used to grade the pathological grading and staging of mice. The grading system included a semiquantitative assessment of three histological characteristics demonstrated in liver H&E staining images (200×): steatosis (0‐3), lobular inflammation (0‐3), and hepatocellular ballooning (0‐2). NAS was calculated as the sum of steatosis, lobular inflammation, and hepatocellular ballooning scores, in which NAS < 3 (non‐nonalcoholic steatohepatitis [NASH]), NAS > 4 (NASH), and NAS between 3 and 4 (possibly NASH).

### Biochemical Analysis

After fasting for 12 h, blood samples were collected from the eyeball and centrifuged (2000 r, 10 min). Serum concentrations of triglycerides (TG), high‐density lipoprotein cholesterol (HDL‐C), and low‐density lipoprotein cholesterol (LDL‐C) were measured by using kits from Jiancheng Bioengineering Institute (A110, A112, A113, China). Serum levels of insulin and MANF were measured by using ELISA kits (ELM‐Insulin, ELM‐MANF, RayBiotech Inc., USA) according to the manufacturer's instructions. To analyze hepatic DNMT1, 150 mg of liver tissue was thoroughly homogenized in 1 mL of PBS. Hepatic DNMT1 levels were measured using ELISA kits (ml038015, Enzyme‐linked Biotechnology, China) and normalized to liver weight.

### RNA Sequencing (RNA‐seq)

Total RNA was extracted from homogenized liver tissue (each group was set with three replicates), which were randomly taken from F1 and F2 female offspring of the CD or HFD group. The detailed procedure of RNA‐seq was performed as previously described.^[^ ^43^ ^]^ Briefly, following RNA extraction, the transcriptomic amplification and library preparation were prepared by using the NEBNext UltraTM RNA Library Prep Kit for Illumina (NEB, USA) following the manufacturer's instructions. Raw data (raw reads) in FASTQ format were processed using in‐house Perl scripts. The analyses of mRNA expression were conducted using the *DESeq* R package (1.18.0). Genes with an adjusted *P* < 0.05 and log twofold change > 1, found by *DESeq*, were differentially expressed. Functional analysis of differentially expressed genes was performed by Nuohe Zhiyuan Technology (Beijing, China).

### Isolation, Culture, and Treatment of Mouse Primary Hepatocytes

Primary hepatocytes were isolated and cultured via a modified two‐step perfusion method.^[^ ^20^, ^44^ ^]^ Briefly, 4‐week‐old female mice of F1 and F2 from the CD and HFD groups were anesthetized with sodium pentobarbital (30 mg kg^−1^, ip). The inferior vena cava was cannulated, and the liver was perfused with 30 mL of prewarmed 37 °C Hank's buffer, containing 50 µM EGTA and 50 mM HEPES, pH 7.4, was then isolated, and the liver capsule was removed. The entire liver was digested for 10 min at 37 °C with 100 CDU mL^−1^ type IV collagenase (C5138, Sigma‐Aldrich, USA). The cell suspension was filtered through a 75 µm strainer, centrifuged at 50 g for 3 min, and washed three times with DMEM. Resuspended hepatocytes were seeded at 2–3 × 10^5^ cells per well in collagen‐coated 6‐well plates. The viability of primary hepatocytes was determined by the trypan blue exclusion test. Cells were cultured with DMEM containing 10% FBS at 37 °C with 5% CO_2_.

To simulate HFD stimulation in vitro and investigate the effect of palmitic acid (PA) on MANF expression, primary hepatocytes were seeded at 2–3 × 10^5^ cells per well and treated with PA, which was dissolved in bovine serum albumin (BSA), and then diluted with complete medium to a final concentration of 0.1, 0.2, 0.3, and 0.4 mM for 48 h. To verify the time of PA‐induced changes in MANF, primary hepatocytes were treated with PA for 12, 24, and 48 h. To verify PA‐induced changes of ER stress and apoptosis, primary hepatocytes were treated with PA at 0.2 mM for 48 h. To simulate ER stress stimulation in vitro and gain more insight into the relationship of ER stress and the glucose metabolic dysfunction, primary hepatocytes were incubated with DMSO or Tg (10^−6^ M), an ER stress inducer, for 48 h.

To investigate the functions of EZH2, DNMT1, H3K27me3, and MANF, primary hepatocytes were treated with PA, GSK126 (S7061, Selleck, USA), a highly selective EZH2 methyltransferase inhibitor, 3‐deazaneplanocin A‐ (S7120, Selleck, USA), a competitive S‐adenosylhomocysteine hydroxylase (SAHH) inhibitor, which depletes EZH2 and the associated H3K27me3, or DC‐05, a DNMT1 inhibitor, for different concentrations at 48 h.^[^ ^39a^ ^]^ DMSO was used as a negative control. The EZH2 and H3K27me3 levels in primary hepatocytes were evaluated by western blot to ascertain the inhibitory effect.

### Sperm Sample Collection

As previously described, mature sperm were isolated from the cauda epididymis of adult male mice belonging to the F0 and F1.^[^ ^8^, ^45^ ^]^ Briefly, sperm were released from the cauda epididymis into 5 mL phosphate‐buffered saline (PBS) maintained at 37 °C for 15 min of incubation, after which they were then filtered with a 40 µm cell strainer to rid the tissue debris. The sperm were then treated with somatic cell lysis buffer (0.1% SDS, 0.5% Triton X‐100 in DEPC H_2_O) for 40 min on ice to eliminate somatic cell contamination, after which the sperm was pelleted by centrifugation at 600 g for 5 min. After removal of suspension, the sperm pellet was resuspended and washed twice in 10 mL of PBS, then pelleted at 600 g for 5 min. Then, the sperm pellet was stored at −80 °C until further processing.

### Quantitative Real‐time Polymerase Chain Reaction (RT‐qPCR)

Total RNA was extracted from liver tissue or primary hepatocytes using TRIZOL reagent (15 596 026, ThermoFisher Scientific, USA) and reverse‐transcribed using the RevertAid First Strand cDNA Synthesis Kit (K1622, ThermoFisher Scientific, USA) according to the manufacturer's instructions. The transcriptional expression of genes was determined by RT‐qPCR using a Bio‐Rad CFX96 PCR System and was analyzed with the 2^−ΔΔCT^ method with 18S as the inner control. All measurements were performed in triplicate. Information regarding the sequences of RT‐qPCR primers is provided in Table S1, Supporting Information.

### Protein Extraction and Immunoblot Analysis

Total proteins of fresh liver tissue, sperm samples, or primary hepatocytes were dissolved using RIPA Lysis Buffer (P0013B, Beyotime, China), and histone extraction was performed according to the manufacturer's instructions (OP‐0006‐10, Epigentek, USA). 50 µg total proteins or 20 µg histones were separated by sodium dodecyl sulfate‐polyacrylamide gel electrophoresis (SDS‐PAGE) and then electrotransferred to the nitrocellulose membranes (HATF00010, Millipore, Germany). The membrane was blocked with 5% nonfat milk for 2 h to remove nonspecific binding and then incubated with primary antibodies (1:1000) overnight at 4 °C. Following being washed with Tris‐buffered saline and Tween 20 (TBST) three times, the membrane was incubated with the horseradish peroxidase‐conjugated secondary antibodies (1:10 000 dilution) at room temperature (RT) for 1 h. The blots were detected by enhanced chemiluminescence (ECL) and captured on the imaging system (Tanon, China). Protein band density was analyzed using Alpha Ease Image Analysis software (version 3.1.2) and normalized to the housekeeping gene β‐ACTIN, or GAPDH. The involved antibodies were included in Table S2 (Supporting Information).

### Nuclear and Cytoplasmic Extraction

The liver tissue was dissected on ice and homogenized in a glass homogenizer to disrupt the cell membrane and release the cytoplasmic contents. According to the manufacturer's kit manual, the proteins in the nuclear and cytoplasmic were extracted by the Nuclear Protein Extraction Kit (P0028, Beyotime Biotechnology, China).

### Immunofluorescence (IF)

8‐cell embryos and primary hepatocyte slides, which were treated with 0.2 mM PA for 48 h, were fixed in 4% paraformaldehyde at RT for 15 min, permeabilized for 10 min in 0.01% Triton X‐100 (85 112, ThermoFisher Scientific, USA) in PBS, and washed twice in PBS. Cells were blocked in 5% BSA in 0.01% Triton X‐100 in PBS for 1 h at RT. The cells were incubated overnight at 4 °C with DNMT1 (68485‐1‐AP, Proteintech, China) and EZH2 (21800‐1‐AP, Proteintech, China). Cells were washed three times with PBS. Alexa Fluor goat anti‐rabbit IgG (Invitrogen, A11037, USA) and Alexa Fluor goat anti‐mouse secondary antibodies (Invitrogen, A32723, USA) were incubated for 1 h at RT. After being washed with PBS, the nuclei of cells were counterstained with DAPI. Fluorescence was visualized under a fluorescent laser scanning microscope (Nikon Eclipse 80i, Japan).

### TUNEL Assay

The apoptosis of liver paraffin was analyzed by the TMR Tunel Cell Apoptosis Detection Kit (G1502, Servicebio, China), following the manufacturer's instructions. Briefly, liver paraffins of mice were permeabilized for 15 min at RT using 1% Triton X‐100 after being washed with PBS. Subsequently, they were incubated with TUNEL staining solution for 1 h in the dark and then washed with PBS. Ultimately, cell nuclei were counterstained with DAPI. Images were captured at 450 nm and 532 nm by fluorescence microscopy (Nikon Eclipse 80i, Japan).

### Flow Cytometry Assays

The early and late stages of apoptotic activity in primary hepatocytes were evaluated using an annexin V‐FITC/propidium iodide (PI) kit (P‐CA‐201, Wuhan Pricella Biotechnology Co., Ltd., China). In brief, cells were collected with EDTA‐free trypsin and then washed twice with PBS. The cell pellet was resuspended in a binding buffer. Annexin V‐FITC and PI were added to the suspensions, respectively. The suspensions were incubated at RT for 15 min in the dark. Then, fluorescence intensities of 1 × 10^4^ cells per sample were detected and recorded by using CytoFLEX (Beckman Coulter, USA).

### siRNA Knockdown and Plasmid Transfection

Primary hepatocytes were seeded in 6‐well plates (5 × 10^5^ cells per well). After 24 h, 75 nmol of siRNA was transfected using lipofectamine 2000 (11 668 019, ThermoFisher, USA) according to the manufacturer's instructions. Negative control siRNA (siNC) and complementary oligonucleotides of the *Manf* gene (si*Manf*) were purchased from GenePharma (Shanghai, China). After transfection for 6 h, cells were harvested after 48 h. RT‐qPCR and immunoblot analysis were used to validate knockdown efficiency prior to this study. The siRNA sequences utilized in gene knockdown experiments are displayed in Table S1 (Supporting Information). In addition, *Manf* CDS was synthesized by Sangon Biotech Company (Shanghai, China) and inserted into the pcDNA3.1 (+) vector using the HindIII or XhoI restriction sites. After being treated with PA for 48 h, 1.5 µg of plasmid were transfected into primary hepatocytes using lipofectamine 2000 in overexpression experiments.

### Intracellular Ca^2+^ Staining

The intracellular calcium ion concentration was measured by Fluo‐3 AM (Invitrogen, Carlsbad, USA). Single primary hepatocytes were obtained and placed on petri dishes, then cultured with 5 µM Fluo‐3 AM in a Ca^2+^‐free solution containing (mM: NaCl, 135; NaH_2_PO_4_, 0.44; KCl, 5.6; NaHCO_3_, 4.2; MgCl_2_, 1; 4‐(2‐hydroxyethyl)‐1‐piperazineethanesulfonic acid (HEPES), 10; Na_2_HPO_4_, 0.42; and glucose, 10; pH 7.4 [adjusted with NaOH]) in the dark for 30 min at 37 °C. Then, a Ca^2+^‐free solution was used to wash the cells three times, and the cells were incubated under the calcium solution (mM: NaCl, 135; KCl, 4.7; CaCl_2_, 1.8; MgCl_2_, 1; HEPES, 10; NaH_2_PO_4_·2H_2_O, 1.2; and glucose, 10; pH 7.4 [adjusted with NaOH]). Single hepatocyte images and traces were recorded for Ca^2+^ responses to Thapsogargin (Tg, 10^−6^ M) using a total internal reflection fluorescence microscopy (TIRFM) electron‐multiplying charge‐coupled device imaging system. All experiments were processed and analyzed using Fiji software (Olympus IX‐71, Japan). The fluorescence intensity was calculated as F/F0, where F0 was fluorescence intensity when the Ca^2+^ activity was stable, and F was the fluorescence intensity when reacting to the drugs. All chemicals were purchased from Sigma‐Aldrich unless otherwise stated.

### ChIP‐qPCR

ChIP assays were performed by using Sample ChIP Plus Enzymatic Chromatin IP Kit (9005, Cell Signaling Technology, USA). Briefly, 37% formaldehyde cross‐linked 150 mg of liver tissue from F0 and F1 mice at RT for 15 min. Glycine then quenched the reaction. The tissue was homogenized with lysis buffer to release the nucleus, and the homogenate was sonicated to shear the DNA into fragments of 150–900 bp. The digested DNA solution was sonicated for 1 min at an amplitude of 15%. The protein/DNA complexes were precipitated with antibodies of H3K27me3 and IgG control overnight. After purification, the immunoprecipitated DNAs were quantified by RT‐qPCR using the primers corresponding to the indicated sites in the promoter regions and normalized to the input. Table S1 (Supporting Information) displays the primer sequences for amplification of gene promoters.

### Co‐Immunoprecipitation (Co‐IP)

Mice (16‐week‐old) of CD‐F0 and HFD‐F0 groups were sacrificed by cervical dislocation. The fresh liver tissue was lysed with modified lysis buffer (50 mM Tris, 150 mMm NaCl, 1 mMm EDTA, 1 mM EGTA, 0.5% sodium deoxyholate, and 1% Triton X‐100) using 1 × protease inhibitor (ab201111, Abcam, UK). Homogenates were centrifuged at 4 °C for 30 min at 13, 200 rpm as previously described.^[^ ^43^ ^]^ Protein samples were pretreated with 20 µL protein A/G agarose beads (sc‐2003, Santa Cruz Biotechnology, USA) for 2 h. After gentle spinning, the supernatant was incubated with DNMT1 or EZH2 antibody at 4 °C. The next day, 50 µL protein A/G agarose beads were added and incubated at RT for 2 h. The beads were washed three times in ice cold lysis buffer, and then eluted and boiled in 2 × loading buffer for western blotting.

### Collection of Mice 8‐Cell Embryos

To get mouse‐fertilized embryos, 4‐week‐old C57BL/6J female mice were super‐ovulated by injection with 5 IU each of pregnant mare serum gonadotropin (PMSG), followed by injection of 5 IU of human chorionic gonadotropin (hCG) (San‐Sheng Pharmaceutical, China) 48 h later.^[^ ^46^ ^]^ The super‐ovulated female mice were mated with 16‐week‐old F0 or F1 male mice. Then, the 8‐cell embryos were collected from the oviducts of the female mice at 67 h post‐hCG injection and were harvested for H3K27me3 immunofluorescence.

### Targeted Bisulfite Sequencing PCR

Genomic DNA was extracted from liver tissues of F0 male mice and F1 female mice in both the CD and HFD groups by the standard phenol/chloroform technique. Then, in accordance with the manufacturer's protocols, DNA was bisulfite converted using the EZ DNA Methylation‐GOLD Kit (D5006, Zymo Research, USA). Following the DNA bisulfite treatment, the fragments were amplified by PCR.^[^ ^39^, ^47^ ^]^ CpG islands of the proximal gene promoter were chosen based on the following criteria: 1) ≥ 200 bp length; 2) ≥ 50% GC content; 3) ≥ 60% ratio of observed/expected dinucleotides in CpG.^[^ ^47^ ^]^ Table S1 (Supporting Information) lists the primer sequences. Illumina HiSeq 2000 sequenced the products after PCR amplification. The average methylation level was calculated using the methylation levels of all measured CpG sites. The methylation level at each tested CpG site was calculated as the percentage of the methylated cytosines over the total tested cytosines.

### Data and Statistical Analysis

All data were expressed as the mean ± SEM and analyzed by GraphPad (Prism 8.4.2) software. Statistical analyses were performed via one‐way/two‐way analysis of variance (ANOVA) followed by the Bonferroni test or a two‐tailed unpaired Student's t‐test. *P* values < 0.05 were considered statistically significant. In each graph, otherwise specifically mentioned, ns, no significance; ^*^, *p* < 0.05; ^**^, *p* < 0.01; ^***^, *p* < 0.001; ^****^, *p* < 0.0001; versus control.

## Conflict of Interest

The authors declare no conflict of interest.

## Author Contributions

Y.S., W.L., and X.Y. are co‐first authors. Conceptualization performed by Y.S.; methodology performed by Y.S., W.L., and L.L.; data analysis performed by Y.S., W.L., D.Z., and X.Y.; data curation performed by Y.S., Y.Z., Y.S., P.Z., Z.Z., B.W., and M.S.; writing the manuscript performed by Y.S. and M.S.; supervision performed by Q.G. and M.S.; funding acquisition performed by M.S.

## Inclusion and Diversity

We support inclusive, diverse, and equitable conduct of research.

## Supporting information



Supporting Information

## Data Availability

The authors confirm that all relevant data are available in the paper and/or its Supplementary Information files or from the corresponding author upon request. Source data are provided with this paper.
