# Supplementary material for: Paternal Obesity‐Induced H3K27me3 Elevation Leads to MANF‐Mediated Transgenerational Metabolic Dysfunction in Female Offspring
Source: Adv Sci (Weinh). 2025 Mar 5;12(16):2415956. doi: 10.1002/advs.202415956 (PMC12021121; doi:10.1002/advs.202415956)
Supplement: Supplementary file 1 — Supporting Information [file ADVS-12-2415956-s001.docx]

**
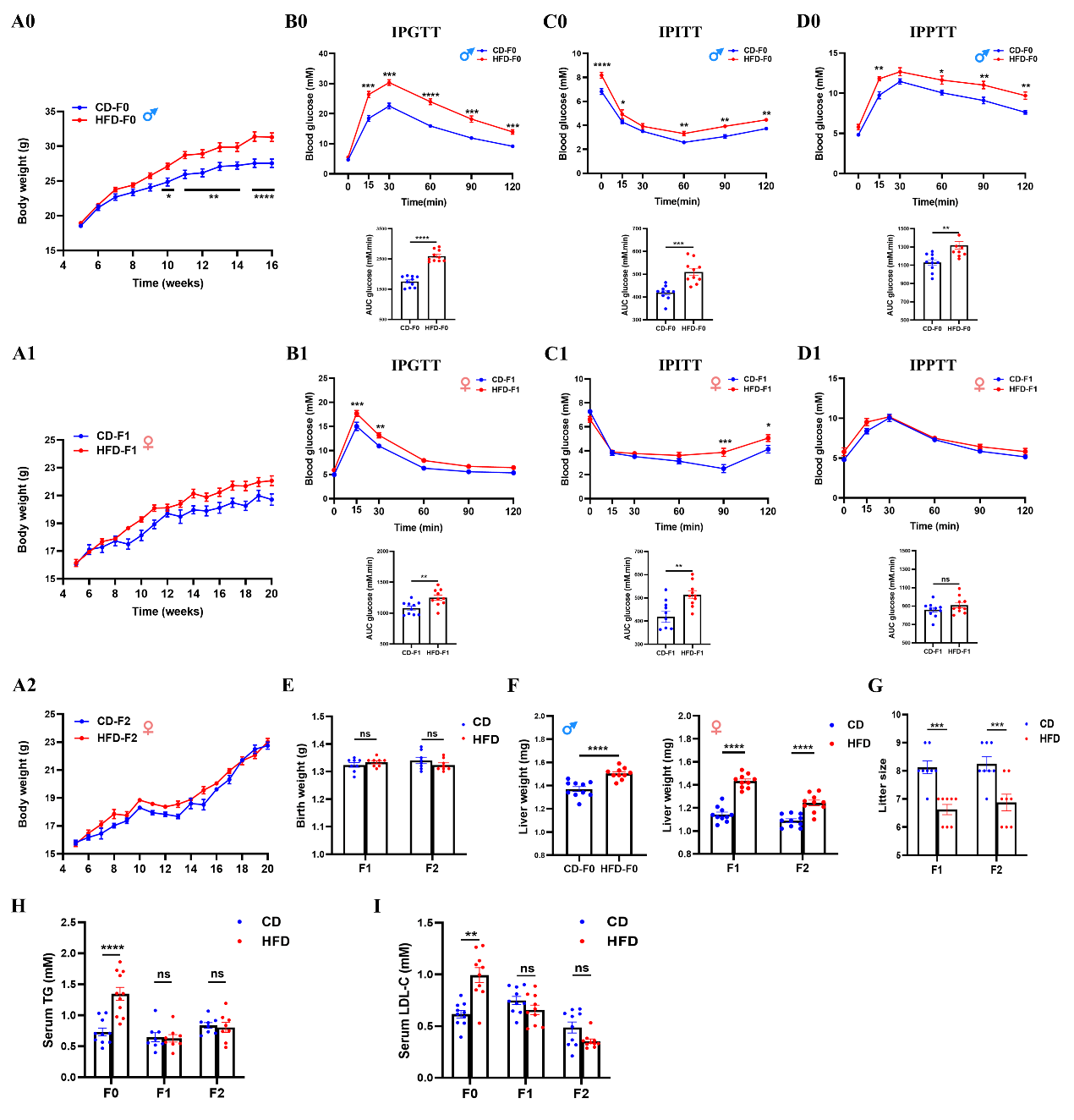
**

**Figure** S**1.** **Paternal HFD exposure impaired** **glucose and fat metabolism and fertility without altering body weight in the female offspring.**

**(A0)** Body weight of F0 male mice with CD or HFD (n = 20-28 mice each group); **(A1-2)** Body weight in F1-F2 female mice (A1: F1 generation, A2: F2 generation, n = 20-28 female mice/group); **(B0-1)** Glucose tolerance test and AUC in F0 male mice or F1 female mice with CD or HFD (B0: F0 generation, B1: F1 generation, n = 10 mice each group); **(C0-1)** Insulin tolerance test and AUC in F0 male mice or F1 female mice with CD or HFD (C0: F0 generation, C1: F1 generation, n = 10 mice each group); **(D0-1)** Pyruvate tolerance test and AUC in F0 male mice or F1 female mice with CD or HFD (D0: F0 generation, D1: F1 generation, n = 10 mice/group); **(E)** Birth weight of the F1 and F2 offspring (randomly selected one pup from each litter, n = 8 each group). **(F)** Liver weight of F0 mice and F1-F2 female mice (n = 10 each group) **(G)** Litter size of the F1 and F2 offspring (n = 8 each group); **(H)** Serum TG levels of F0-F2 generation (n = 10 each group); **(I)** Serum LDL-C levels of F0-F2 generation (n = 10 each group). Data are mean±SEM. ns, no significance; * *p*< 0.05; ** *p* < 0.01; *** *p*< 0.001; **** *p*< 0.0001 (two-tailed t-test or two-way ANOVA).

**
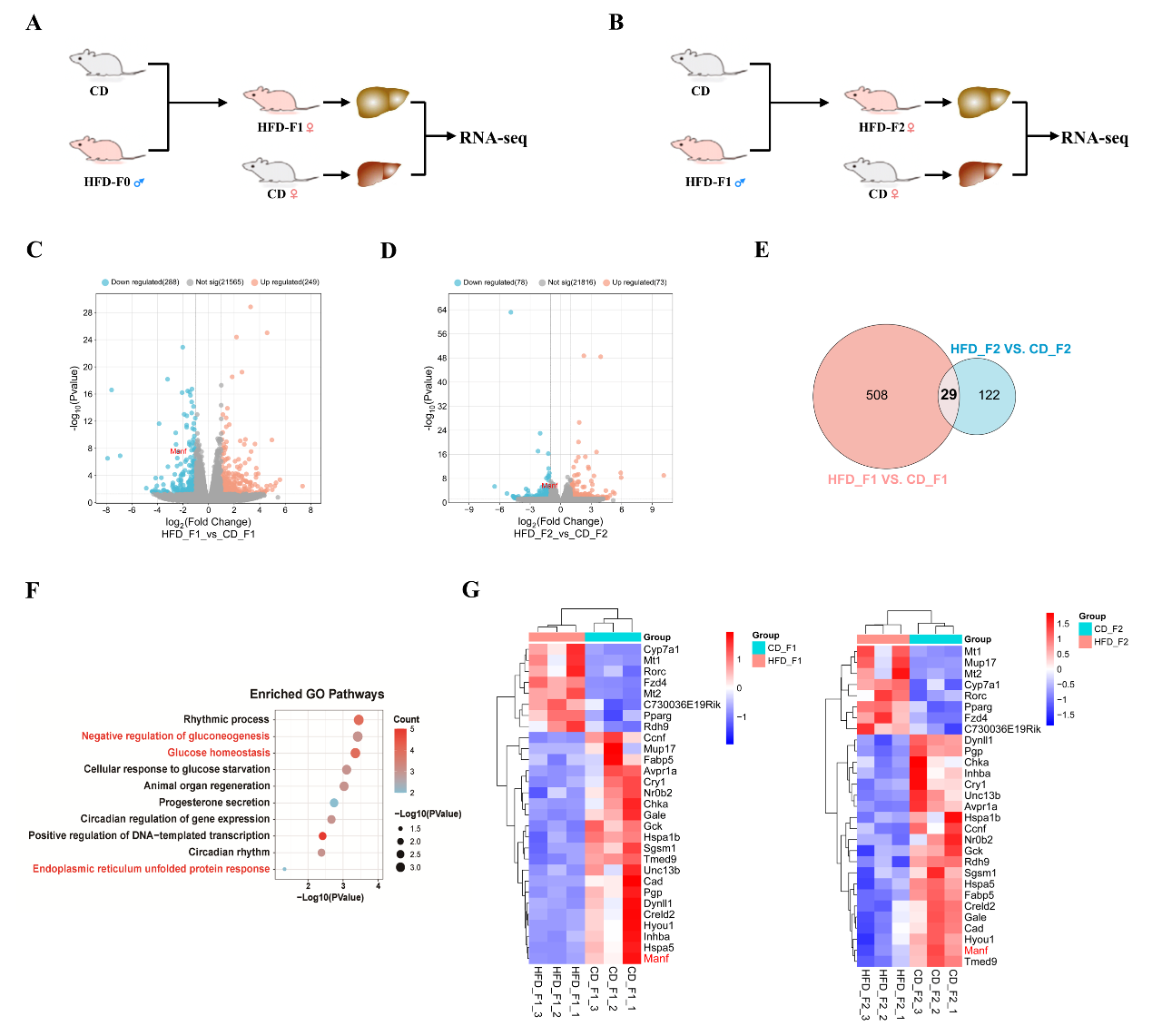
**

**Figure** S**2. Paternal obesity leads differential expression of mRNA in the liver of female offspring at F1-F2 generation.**

**(A-B)** CD-F0 or HFD-F0 male mice were mated with 8-week-old unexposed female mice to generate the F1, and the same mating scheme was used to generate the F2. The left lateral lobe of liver in adult female mice was isolated for RNA-sequencing; **(C)** Volcano plot showing the differential expression genes between CD-F1 and HFD-F1 female mice. Red points denote the upregulated genes (249 genes), and blue points indicate the downregulated genes (288 genes) (n = 3 each group); **(D)** Volcano plot showing the differential expression genes between CD-F2 and HFD-F2 female mice. Red points denote the upregulated genes (73 genes), and blue points indicate the downregulated genes (78 genes) (n = 3 each group); **(E)** Venn diagram of DEGs in F1 RNA-seq results and DEGs in F2 RNA-seq results; **(F)** The top 10 biological processes in GO analysis of these 29 DEGs; **(G)** Heatmap of these 29 DEGs in the liver of F1 and F2 female offsprings (n = 3 each group).


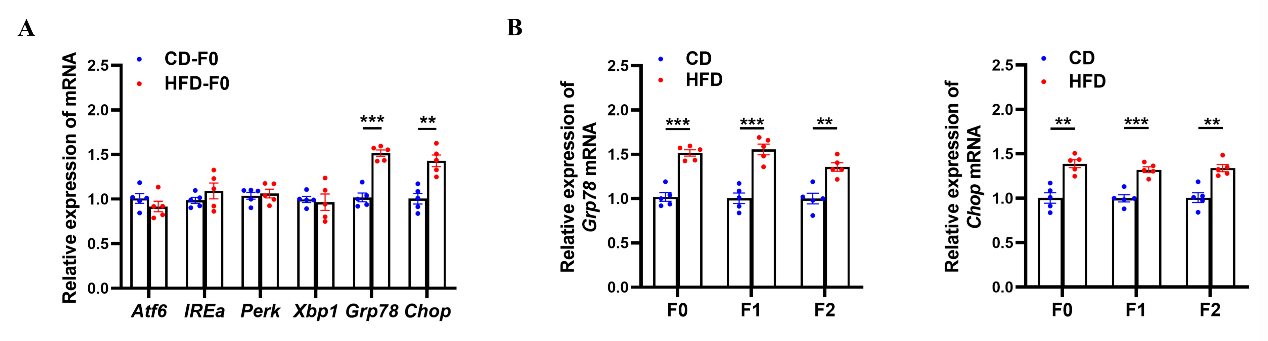


**Figure** S**3. Paternal obesity altered UPR signaling pathways associated with transgenerational glucose metabolic dysfunction.**

**(A)** RT-qPCR verified the gene expression of UPR signaling pathways in the liver of CD-F0 and HFD-F0 (n = 6 each group); **(B)** The mRNA levels of *Grp78* and *Chop* in the liver of F0-F2 generation (n = 6 each group). Data are mean±SEM. ns, no significance; * *p*< 0.05; ** *p* < 0.01; *** *p*< 0.001 (two-way ANOVA).


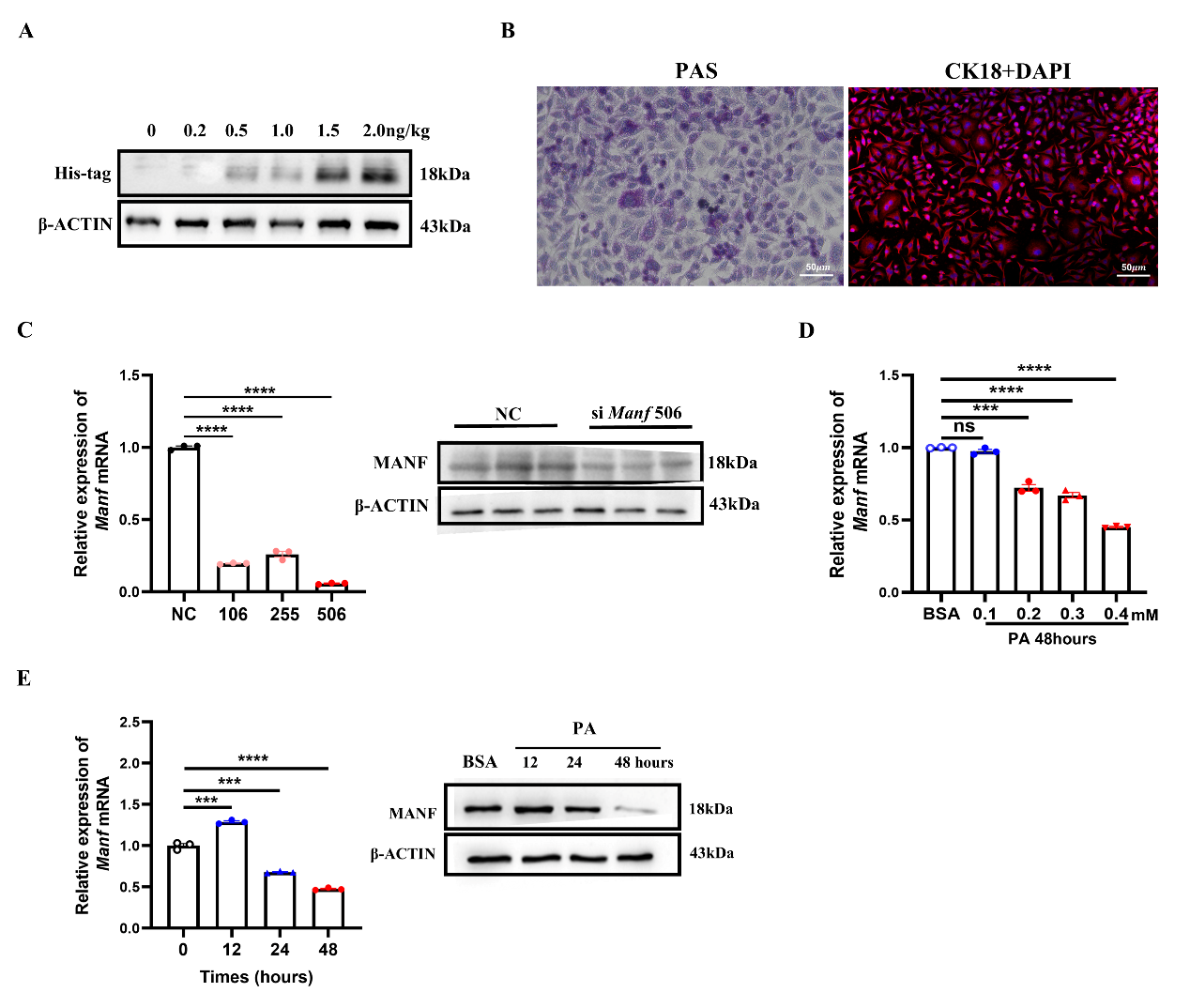


**Figure** S**4. Effect of PA on the expression of MANF in primary hepatocytes.**

**(A**) The protein level of His-tag in the liver of F1 female offspring after intravenously injected with different doses of hMANF for 2 weeks; **(B)** Primary hepatocytes were identified with PAS staining and CK18 (red). Nuclei are labeled by DAPI (blue); **(C)** The expression of MANF after siRNAs knockdown (n = 3 each group); **(D)** The mRNA level of *Manf* was determined after incubating with different doses of PA for 48 hours (n = 6 each group); **(E)** The mRNA and protein levels of MANF were determined after being treated with PA at 0.2 mM for different times (n = 6 each group). Data are mean±SEM. ns, no significance; *** *p*< 0.001; **** *p*< 0.0001 (one-way ANOVA).


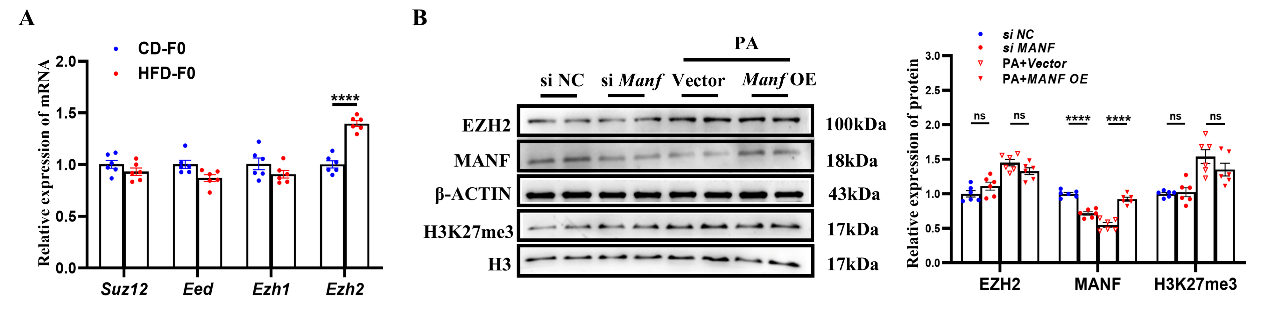


**Figure S5. MANF did not affect the expression of EZH2 and H3K27me3.**

**(A)** The mRNA level of *Suz12, Eed, Ezh1,* and *Ezh2* in the liver of CD-F0 and HFD-F0 was determined by RT-qPCR (n = 6 each group); **(B)** Protein levels of EZH2, H3K27me3, and MANF in primary hepatocytes of different groups (n = 6 each group). Data are mean±SEM. ns, no significance; * *p*< 0.05; ** *p* < 0.01; *** *p*< 0.001.

**
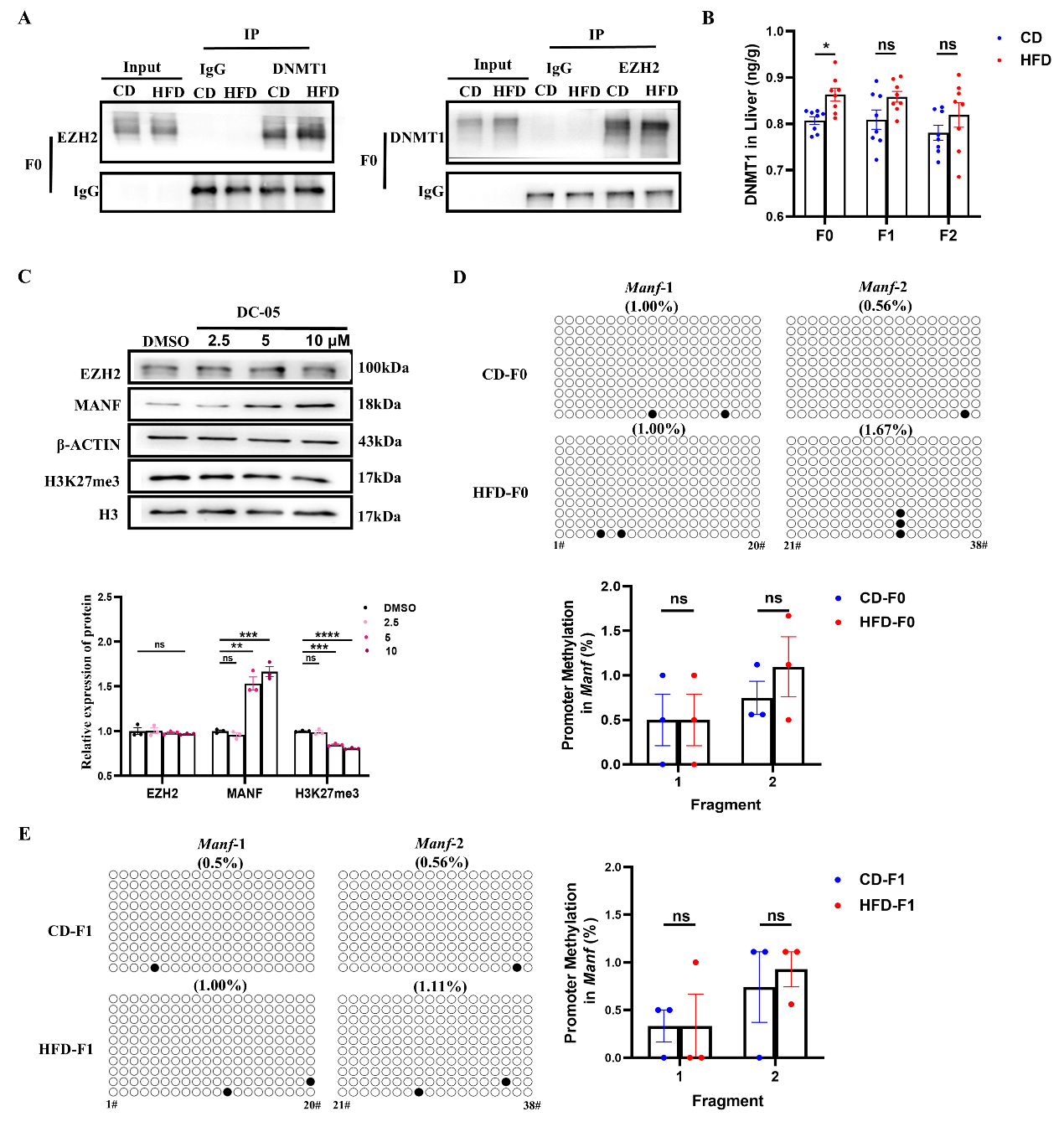
**

**Figure S6. EZH2 recruits DNMT1 to mediate H3K27me3 modification, which results in the downregulation of MANF.**

**(A)** DNMT1 antibody, EZH2 antibody, and control IgG antibody were used for immunoprecipitation of the F0 liver tissue. DNMT1-interacting proteins were examined by western blotting with anti-EZH2. EZH2-interacting proteins were examined by western blotting with anti-DNMT1. The representative images were shown; **(B)** DNMT1 levels in the liver of F0-F2 generation between CD and HFD groups (n = 10 each group); **(C)** Protein levels of EZH2, H3K27me3, and MANF in primary hepatocytes after incubating with different doses of DC-05 for 48 hours (n = 3 each group); **(D)** DNA methylation on the *Manf* promoter in the liver of F0 mice. DNA methylation at CpG sites was quantified using bisulfite sequencing. Filled circle, methylated; open circle, unmethylated (n = 3 each group). **(E)** DNA methylation on the *Manf* promoter in the liver of F1 female mice. DNA methylation at CpG sites was quantified using bisulfite sequencing. Filled circle, methylated; open circle, unmethylated (n = 3 each group). Data are mean±SEM. ns, no significance; * *p*< 0.05; ** *p* < 0.01; *** *p*< 0.001; **** *p*< 0.0001 (two-way ANOVA).


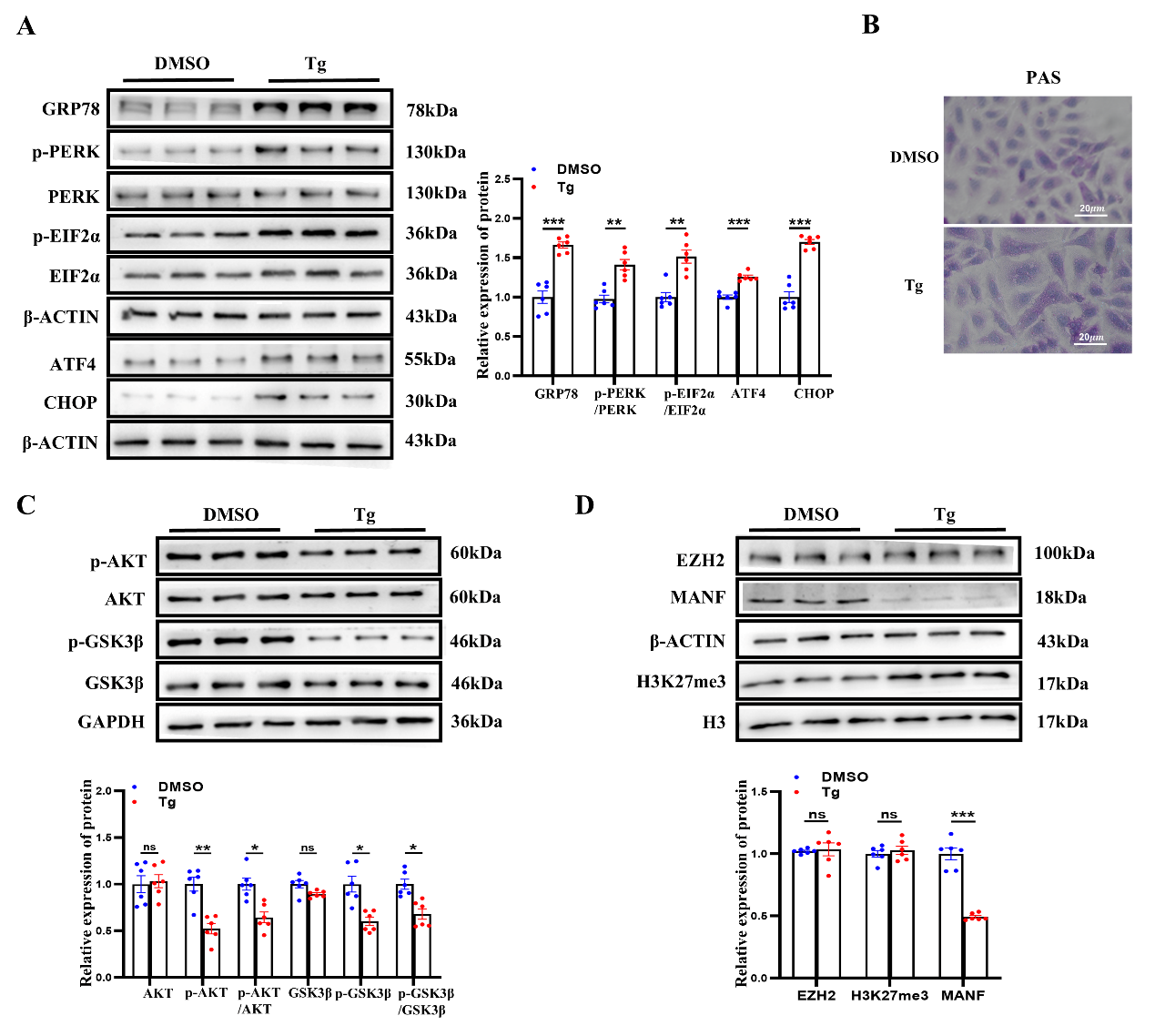


**Figure S7. Tg-induced ER stress decreases MANF *in* *vitro*, leading to glucose homeostasis disorders*.***

**(A)** Protein levels of the GRP78-PERK-EIF2A-ATF4-CHOP pathways in primary hepatocytes of DMSO and Tg groups (n = 6 each group); **(B)** PAS staining of primary hepatocytes in different groups (n = 3 each group); **(C)** Protein levels of AKT, p-AKT, p-AKT/AKT, GSK3β, p-GSK3β, p-GSK3β/GSK3β in primary hepatocytes of different groups (n = 6 each group); **(D)** Protein levels of the EZH2-H3K27me3-MANF pathway in primary hepatocytes of different groups (n = 6 each group); Data are mean±SEM. ns, no significance; * *p*< 0.05; ** *p* < 0.01; *** *p*< 0.001 (two-way ANOVA).

**Supplemental Table 1. List of primers used in this study.**

| **Primer Sequence for qRT-PCR (5’-3’)** | | |
| --- | --- | --- |
| Gene name | Forward primer | Reverse primer |
| *Manf* | AGCTTCTTCAGGTCCACTGTG | AGATCATCAATGAGGTGTCGAAG |
| *Atf6* | CAGCTGATGGCTGTCCAGTA | GCGTCAAAGAAGCCTTGGTA |
| *Ire1α* | TGTTTGTCTCGACCCTGGAT | ACTGCCATCATTGGGATCTG |
| *Perk* | ATGGGAAAAAGCAGTGGGAT | TGGAAGAGGTCTCCATCCAG |
| *Xbp1* | TTAAGAACACGCTTGGGAATG | ATGTTCTGGGGAGGTGACAA |
| *Grp78* | ATGAAGGAGACTGCTGAGGC | CAGCATCTTTGGTTGCTTGTC |
| *Chop* | TGAGGATCTGCAGGAGGTCCT | GCTCCTCTGTCAGCCAAGCT |
| *Suz12* | TCGAAATGGACCGGTAAAGA | AGAAACTCCGACATGCTTGC |
| *Eed* | TATGTTGATTGTGTGCGATGG | CAGCATACAATGGCATTTTCAC |
| *EZh1* | AAAGAGGACCTGCCGGTAAC | AATGGCGCTGAAGATCATGT |
| *Ezh2* | GCCAAATCTGTTCAGAGGGA | ATGTGTTGGGTGTTGCATGG |
| *18S* | CTCAACACGGGAAACCTCAC | CGCTCCACCAACTAAGAACG |
| **siRNA targeting sequence** | | |
| Gene name | Sense | Antisense |
| *Manf* | GACUAUAUCCGGAAGAUAATT | UUAUCUUCCGGAUAUAGUCTT |
| Negative  Control (NC) | UUCUCCGAACGUGUCACGUTT | ACGUGACACGUUCGGAGAATT |
| **Primer Sequence for Chip-PCR (5’-3’)** | | |
| Position | Forward primer | Reverse primer |
| *Manf* -1.0kbp | TCATGGCATCTGGCATCAC | AGAGAATCGGTTGGTAAGTAGC |
| *Manf* -0.8kbp | ATTCTCTTTGCCTCTTGCTTCA | ACTTACCATCTCCTCCCTCTTT |
| *Manf* -0.4kbp | GACAGATTGAAGGCTGAAGCA | TTAGCGATTACAGGAAGGCATC |
| *Manf* +0.4kbp | GAATGCCGAGTCTTGGTTCC | TTAAGAGGTTCTGCCGTCTGA |
| *Manf* +0.8kbp | TCTTGGAGACACGAGGATGG | TGATTTCACTGAATGGGAGCAT |
| *Manf* +1.0kbp | TCACATCACAGCAGTCAAGGG | TGTGCCAGTGTCCCAAGAG |
| *Manf* +2.0kbp | TGTGGAGAAGGGATAAGCAGC | TCCTTGATGAGCAGGTATGTTG |
| **Primer Sequence for BSP (5’-3’)** | | |
| Gene name | Forward primer | Reverse primer |
| *Manf*-1 | TGGTTTTTATTTGGAAGGTAGATTA | AAATTAAAACAACTACTTTAACTACTTACC |
| *Manf*-2 | TAATGAGGATGTAGTATATGGGTGG | CCRCACCTTCACAATCTCCT |

**Supplemental Table 2. List of antibodies used in this study.**

| **Antibodies** | **Source** | **Identifier (**Cat.No:**)** |
| --- | --- | --- |
| AKT antibody | Cell Signaling Technology | 9272 |
| phospho-AKT (Ser473) antibody | Cell Signaling Technology | 4060 |
| GSK3β antibody | Cell Signaling Technology | 9315 |
| phospho-GSK3β (Ser9) antibody | Cell Signaling Technology | 9323 |
| CHOP antibody | Cell Signaling Technology | 12456 |
| MANF antibody | Abcam | ab316935 |
| Ezh2 antibody | Proteintech | 21800-1-AP |
| GRP78 antibody | Proteintech | 11587-1-AP |
| ATF4 antibody | Proteintech | 10835-1-AP |
| EIF2α antibody | Proteintech | 11233-1-AP |
| phospho-EIF2α (Ser51) antibody | Proteintech | 28740-1-AP |
| PERK antibody | Immunoway | YT3666 |
| phospho-PERK (Thr981) antibody | Immunoway | YP1055 |
| H3K9me3 antibody | Active Motif | 39065 |
| H3K27me3 antibody | Active Motif | 39055 |
| Beta Actin Monoclonal antibody | Proteintech | 66009-1-Ig |
| GAPDH Monoclonal antibody | Proteintech | 60004-1-Ig |
